# Comparative Phytochemical Profiles of Medicinal Plants Used for Wound Treatment: Insights From Wild and Hydroponically Cultivated Species in Lugazi Diocese, Uganda

**DOI:** 10.1002/cbdv.202503018

**Published:** 2026-01-17

**Authors:** Ivan Kahwa, Christina Seel, Hilda Ikiriza, Maria Kulosa, Susan Billig, Claudia Wiesner, Anke Weisheit, Olivia Harriet Makumbi, André Gerth, Leonard Kaysser

**Affiliations:** ^1^ Department of Pharmaceutical Biology, Faculty of Medicine, Institute For Drug Discovery Leipzig University Leipzig Germany; ^2^ Department of Pharmacy, Faculty of Medicine Mbarara University of Science and Technology Mbararam Uganda; ^3^ Pharm‐Biotechnology and Traditional Medicine Centre Mbarara University of Science and Technology Mbarara Uganda; ^4^ Department of Biology Faculty of Science Mbarara University of Science and Technology Mbarara Uganda; ^5^ Institute of Analytical Chemistry, Faculty of Chemistry Leipzig University Leipzig Germany; ^6^ Lugazi Rural Finance Development Trust, Namagunga Lugazi Uganda; ^7^ Independent Researcher, Fuerstenweg Grimma Germany

**Keywords:** *Centella asiatica*, *Conyza sumatrensis*, hydroponics, *Justicia betonica*, phytochemical profiling

## Abstract

Medicinal plants such as *Centella asiatica*, *Conyza sumatrensis*, and *Justicia betonica* are widely used in Uganda for traditional wound healing. However, the impact of cultivation conditions on their therapeutic potential remains poorly understood. This study compared the phytochemical profiles and bioactivities of hydroponically cultivated and wild‐collected material of these species from Lugazi Diocese, Uganda. Extracts were prepared using ethanol, methanol, and water, and analyzed by thin‐layer chromatography (TLC), high‐performance liquid chromatography (HPLC‐UV), headspace gas chromatography–mass spectrometry (HS‐GC–MS), and liquid chromatography–tandem mass spectrometry (LC–MS/MS). TLC and HPLC‐UV indicated terpenoids, flavonoids, and steroids, while HS‐GC–MS revealed predominantly monoterpenes and sesquiterpenes. LC–MS/MS annotated flavonoids, including quercetin‐3‐O‐glucuronoside, kaempferol‐3‐O‐rutinoside, and kaempferol, as well as triterpenoids such as asiatic acid and katononic acid. Antibacterial activity was evaluated against *Bacillus subtilis*, *Escherichia coli*, *Pseudomonas fluorescens*, and *Saccharomyces cerevisiae* using the agar well diffusion method. Anti‐inflammatory effects were assessed by IL‐6 and IL‐8 secretion, and cytotoxicity by MTT assay. Ethanol and methanol extracts exhibited moderate antibacterial activity, while aqueous extracts of wild *C. asiatica* and hydroponic *C. sumatrensis* significantly reduced IL‐6 secretion. No cytotoxic effects were detected. These findings suggest hydroponic cultivation preserves essential phytochemicals and bioactivities, supporting sustainable production of medicinal plants for therapeutic applications.

## Graphical abstract

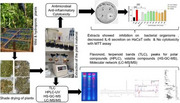

## 1 Introduction

The process of wound healing involves intricate biological mechanisms that are vital to rebuilding the skin after injuries, such as cuts or other accidents. It occurs through several stages in the order of hemostasis, inflammation, proliferation, and tissue remodeling [1, 2]. In developing countries such as Uganda, injuries mainly arise from traffic‐related accidents, burns due to fires and cuts from sharp objects such as knives or pangas, among others [3].

Many people living in rural areas rarely use conventional medication, such as antibiotics, to treat wounds, causing public health concerns through increased mortality rates [4, 5].

The widespread use of alternative herbal remedies, particularly in Uganda's rural communities, is reportedly mainly due to limited access to healthcare and affordable modern medicines [6, 7, 8, 9]. These traditional medicinal plants contain phytochemicals, such as phenolic compounds, flavonoids, tannins, terpenoids and alkaloids with antimicrobial, anti‐inflammatory, and antioxidant properties, which could support the wound healing process [10, 11, 12, 13]. Typically, the plant material used comes from wild collections, for example, in forests and bushes [9].

However, unsustainable harvesting practices in the search for herbal remedies can have profound impacts on the environment and have already led to the extinction of important medicinal plants [14]. To overcome such problems, there is an urgent need to explore conservation strategies and alternative cultivation methods. Hydroponics offers a promising solution, as the controlled growth conditions can be easily optimized to increase the yield of medicinal plant material, ensuring consistent quality and quantity of bioactivity and chemical composition. Hydroponic cultivation systems have been shown to enhance biomass production and the synthesis of secondary plant metabolites compared to conventional agricultural methods [15]. For instance, a recent review article conducted by Atherton and Li [16] has shown elevated levels of metabolites such as glycyrrhizin in *Glycyrrhiza uralensis* Fisch. and hydroponic cultivation increased essential oil yields in Aloysia and root metabolite yields in *Valeriana*, respectively.

In this cultivation technique, medicinal plants are fed with specific nutrients such as nitrogen, phosphorus, potassium, and micronutrients, containing fertilizers [16, 17, 18]. In Uganda, hydroponics systems have so far mainly been applied to small‐scale urban farming for vegetables such as lettuce in non‐greenhouse conditions [19]. To the best of our knowledge, no study has been conducted in Uganda on the use of hydroponic systems to cultivate medicinal plants in a greenhouse. Here, we compare the metabolic profiles of *Centella asiatica*, *Conyza sumatrensis*, and *Justicia betonica*, sourced from the wild and grown in a hydroponic system, using different analytical techniques and examining their respective antimicrobial and anti‐inflammatory activities. The three plants were chosen based on a review of existing literature on medicinal plants used for wound healing. For example, *C. asiatica* is well‐known for its wound healing and ulcer treatment properties [20]. In Uganda, traditional healers also use *C. sumatrensis* for wound care [21]. *J. betonica* is traditionally used for its anti‐inflammatory, analgesic and antimicrobial effects [22]. Therefore, we want to contribute to the existing knowledge of their use in wound treatment among local communities in Lugazi Diocese.

## 2 Results and Discussion

### 2.1 Mini Ethnobotanical Survey Results

Using a semi‐structured questionnaire, we collected responses from participants regarding the application of *C. asiatica*, *C. sumatrensis*, and *J. betonica* for wound treatment.

#### 2.1.1 Demographic Characteristics of the Respondents

Eighteen respondents participated in the interviews, with the majority (50%) aged between 40 and 60 years. Females constituted a significant proportion (88.9%) of the population, and farming was the primary source of income for these individuals (77.8%). More than half of the respondents had a low educational background, with 55.6% having completed only primary education. In addition, 16.7% were illiterate, while another 16.7% had completed secondary education, and 11.1% held college degrees, as illustrated in Table 1.

**TABLE 1:** Demographic characteristics of the respondents in the ethnobotanical survey.

Parameter	Group	Number	%
Age	≤ 20	01	5.60
	20–40	03	16.7
	40–60	09	50.0
	≥ 60	05	27.8
Occupation	Farmer	14	77.8
	Trader	01	5.60
	Teacher	02	11.10
	Student	01	5.60
Sex	Male	02	11.10
	Female	16	88.90
Education level	No formal education	03	16.70
	Primary	10	55.60
	Secondary	03	16.70
	College	02	11.10

#### 2.1.2 Types of Wounds Treated

The participants reported that cuts were the most common type of wounds treated (33%), with ulcers and internal wounds following closely at 31%. Wounds caused by accidents accounted for 16% of cases. Other types of wounds addressed with medicinal plants, though to a lesser degree, included skin and ear injuries (Figure S1A). These results highlight the importance of herbal medicine for wound management in situations where traditional healthcare is unavailable, as evidenced by recent ethnobotanical studies on wound treatment in Uganda [5].

#### 2.1.3 Preparation Methods for Medicinal Plant Remedies

Among the respondents, 60% reported boiling parts of plants to make decoctions, while 23% used leaves directly on wounds. The smallest group, 17%, prepared poultices by crushing fresh leaves and applying them to wounds, as illustrated in Figure S1B. In some cases, the decocted plant materials can be applied directly to the wound [23], thus for the extraction of our plant materials, we adopted decoction as an extraction method.

#### 2.1.4 Mode of Administration of the Medicinal Plants

Most respondents (64%) took the prepared remedies orally, while 36% reported applying them directly to their wounds (Figure S1C). These two methods of administration are often used concurrently to treat both internal wounds, such as ulcers, and external wounds, including cuts. Thereby, smearing is conducted for external wounds, whereas the decoction or juice is taken in [24]. Furthermore, oral administration has long been preferred for internal wounds, as it is reported to reduce inflammation [25].

### 2.2 Extractive Yields for the Medicinal Plants in Different Solvents

Cultivation methods are favored if they can provide a consistent supply of medicinal plants, with metabolite content and yield being the main considered factors [16]. In our study, this is represented by the percentage yields of the aqueous and alcoholic leaf extracts of the three plants. As shown in Table 2, the yields of *C. asiatica* ethanolic extracts (CAEEs) were higher with hydroponic samples (30.7%) compared to wild samples (21.3%). In contrast, the yields for *C. asiatica* aqueous extracts (CAAEs) from hydroponic environments were lower (9.6%) than those from wild plants (15.6%). There was a significant increase in the yield of *J. betonica* methanolic extracts (JBMEs) from hydroponics (16.2%) compared to the wild extract (8.9%). Conversely, *J. betonica* aqueous extracts exhibited similar yields (7.7% for wild and 9.6% for hydroponic). Finally, the yields of *C. sumatrensis* ethanolic extracts (CSEEs) were higher for hydroponic plants (13.4%) than for wild plants (11.1%). However, the aqueous extracts showed a slight decline in yield from hydroponic samples (6.2%) compared to wild samples (8.0%). These results suggest that hydroponic cultivation may enhance yields of certain extracts, particularly those obtained through ethanolic extraction, while having a less significant impact on aqueous extracts. These trends corroborate earlier work showing that cultivation under optimized conditions often enhances the biomass or extractables of medicinal plants [26]. No literature was explicitly found comparing the yields of these species between wild and hydroponic conditions. Still, our data align with general observations that cultivation can either enhance or dilute phytochemical pools, depending on the species and solvent. Most phytopharmaceutical companies are currently adopting hydroponic cultivation to produce plant secondary metabolites [16]. The simple controlled system established at Namagunga demonstrated that it is possible to cultivate the three plants within controlled 90‐day cycles, with water renewal every 2 weeks, using locally constructed greenhouses and nitrogen–phosphorus–potassium (NPK)‐based nutrient regimes (*C. asiatica*/*J. betonica*: 17–17–17, 0.5 g/L; *C. sumatrensis*: 19–19–19, 1 g/L). Reported hydroponic benchmarks indicate yields ranging from 0.1 to 0.3 kg of dry weight (DW) per square meter per cycle. This equates to approximately 5–45 g of extract per square meter, assuming a recovery rate of 5%–15% [15, 16]. In this study, *C. asiatica* could be harvested multiple times, *C. sumatrensis* was suitable for short cropping cycles, and *J. betonica* allowed for staggered pruning. Thus, when combined with controlled nutrient regimes, automation, and validated process controls, hydroponic cultivation offers a technically and economically feasible method to produce standardized extracts from these plants.

**TABLE 2:** Comparative percentage yield for ethanolic, methanolic and aqueous extracts of *Centella asiatica*, *Justicia betonica*, and *Conyza sumatrensis*.

Extraction solvent	Cultivation method	Percentage yield (% w/w)		
		*C. asiatica*	*J. betonica*	*C. sumatrensis*
Ethanolic/methanolic	Wild	21.3	8.90	11.1
	Hydroponics	30.7	16.2	13.4
Aqueous	Wild	15.6	7.70	8.00
	Hydroponics	9.60	9.60	6.20

*Note*: Values show single‐determination yields under standardized conditions with fixed solvent ratio, temperature, and duration. Due to limited biomass, no replicates were performed, and therefore, no statistical data are provided.

### 2.3 Comparison of Thin‐Layer Chromatography and HPLC Phytochemical Profiles for Wild vs. Hydroponically Grown Samples

#### 2.3.1 Thin‐Layer Chromatography Profiling of the Samples

To provide an initial overview of the phytochemical composition of our plant samples collected in the wild and grown under hydroponic conditions, we prepared thin‐layer chromatography (TLC) profiles of the different extracts. TLC analysis detected the preliminary presence of phytochemical groups, visualized at 254, 366 nm, and after derivatization with *p*‐anisaldehyde sulfuric acid, as shown in Table 3. The CAEE from the wild displayed multiple bands across all visualized and detection modes, with notable *R*_f_ values at 0.20, 0.54, 0.84, and 0.94. In contrast, CAAE showed fewer bands, especially at 254 nm. Hydroponic CAEE samples exhibited *R*_f_ bands at 0.10, 0.48, 0.66, and 0.81 under 254 nm light and revealed up to 14 bands after derivatization (e.g., at 0.16, 0.35, 0.72, and 0.94). Meanwhile, CAAE from hydroponics showed minimal banding, indicating a lower phytochemical diversity.

**TABLE 3:** Comparative *R*_f_ values for aqueous and ethanolic *Centella asiatica* and *Conyza sumatrensis*; then methanolic and aqueous extracts of *Justicia betonica* at 254 and 366 nm.

Wavelength	Extraction solvent	Cultivation	*C. asiatica*	*J. betonica*	*C. sumatrensis*
254 nm	Ethanol/methanol	Wild	ND	ND	0.15, 0.43,0.66
		Hydroponics	0.10, 0.48, 0.66, 0.77, 0.81	0.12, 0.46, 0.78, 0.82	0.22, 0.29, 0.67, 0.81
	Aqueous	Wild	ND	ND	0.14, 0.52, 0.77
		Hydroponics	0.04, 0.07, 0.42	0.04, 0.09, 0.12	0.08, 0.12, 0.41
366 nm	Ethanol/methanol	Wild	**0.06**, **0.08**, 0.20, 0.38, **0.44**, **0.54**, **0.72**, **0.84**, 0.89, 0.94	0.08, 0.10, 0.20, 0.39, 0.54, 0.59, 0.76, 0.81,0.87, 0.95	0.02, 0.05, 0.10, 0.15, 0.28, 0.39, 0.43, 0.71, 0.77, 0.82, 0.86, 0.92
		Hydroponics	**0.06**, **0.10**, 0.13, 0.23, 0.35, **0.43**, 0.52, **0.55**, 0.63, **0.7**1, 0.78, **0.83**	0.01, 0.07, 0.12, 0.16, 0.25, 0.30, 0.38, 0.47, 0.53, 0.59, 0.64, 0.69, 0.73, 0.76, 0.83	0.06, 0.10, 0.27, 0.31, 0.36, 0.45, 0.53, 0.57, 0.63, 0.66, 0.71, 0.76, 0.80
	Aqueous	Wild	0.07, 0.11, 0.26, 0.62, 0.93	0.06, 0.16, 0.42	0.06, 0.93
		Hydroponics	0.48	0.52, 0.84	0.09, 0.41, 0.74
Visible	Ethanol/methanol	Wild	0.16, 0.20, 0.28, 0.36, 0.41, 0.54, 0.60, 0.64, 0.74, 0.79, 0.84, 0.90, 0.94	0.18, 0.23, 0.28, 0.33, 0.38, 0.49, 0.56, 0.64, 0.73, 0.79, 0.85, 0.93	0.14, 0.27, 0.35, 0.41, 0.51, 0.56, 0.63, 0.78, 0.84, 0.90
		Hydroponics	0.16, 0.21, 0.32, 0.35, 0.39, 0.49, 0.55, 0.61, 0.72, 0.76, 0.82, 0.85, 0.90, 0.94	0.15, 0.20, 0.33, 0.38, 0.49, 0.54, 0.63, 0.73, 0.78, 0.83, 0.89, 0.94	0.2, 0.36, 0.50, 0.75, 0.83, 0.90
	Aqueous	Wild	0.44, 0.54, 0.60, 0.69, 0.78, 0.83	0.23, 0.61, 0.73	0.18, 0.64, 0.74, 0.91
		Hydroponics	0.57, 0.75, 0.84	0.30, 0.59, 0.71, 0.92	0.13, 0.56, 0.77, 0.90

Abbreviation: ND, not detected.

In JBME, wild samples exhibited broad banding with key *R*_f_ values of 0.20, 0.39, 0.73, and 0.93 across the various visualization methods. Following derivatization, JBME derived from hydroponics showed a similar diversity, with bands at 0.20, 0.33, 0.49, and 0.94. JBAE from both sources was less complex but consistently displayed bands at 0.42 and 0.73.

In CSEE, the wild‐type displayed significant bands with *R*_f_ values of 0.15, 0.43, and 0.66 at 254 nm, and 0.28, 0.39, 0.71, and 0.92 at 366 nm. Derivatization identified extra bands at 0.35, 0.56, 0.78, and 0.90. Similarly, CSEE from hydroponics demonstrated comparable diversity, featuring prominent bands at 0.27, 0.45, 0.66, and 0.83. In contrast, *C. sumatrensis* aqueous extracts (CSAEs) from both sources exhibited limited resolution, with consistent bands observed at 0.74 and 0.90 following derivatization.

**FIGURE 1:**
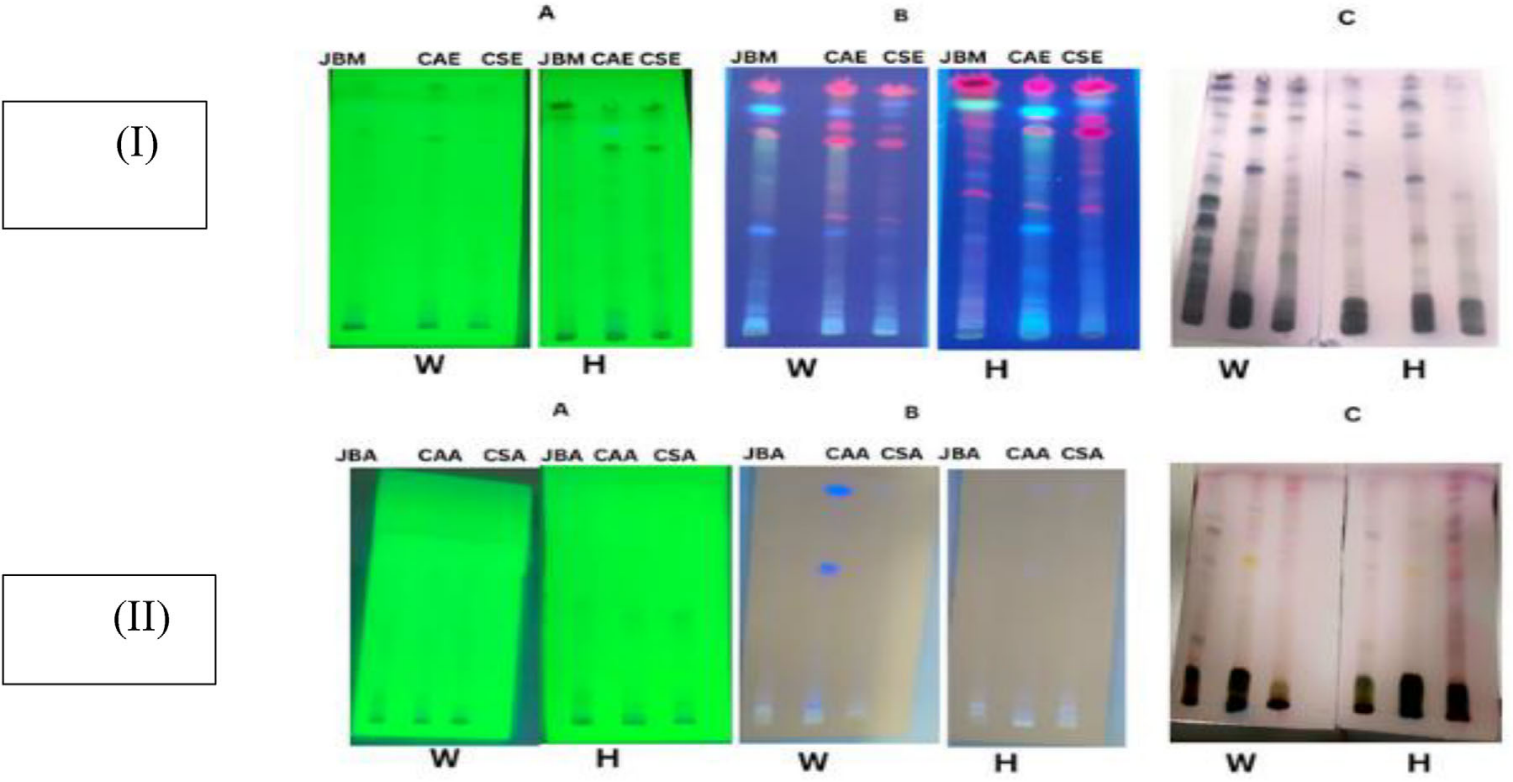
A comparison of TLC profiles for wild (W) and hydroponic (H) extracts of *Centella asiatica* (CAE), *Justicia betonica* (JBM), and *Conyza sumatrensis* (CSE). Key bands associated with flavonoids and terpenoids appeared at *R*_f_ values of 0.20, 0.54, 0.84 in wild CAE, and at 0.10, 0.48, 0.66 in hydroponic CAE. Hydroponic extracts showed slightly fewer spots (up to 12 bands) compared to wild samples (up to 16 bands), suggesting a slight decrease in metabolite diversity. The most intense bands were seen in ethanolic and methanolic extracts. Detection was carried out under UV at 254/366 nm and after derivatization with *p*‐anisaldehyde–H_2_SO_4_.

The TLC plates showed blue, purple, yellow, and grey bands, indicating the presence of phenols, terpenes, sugars, and steroids in both wild and hydroponic samples (Figure 1). Significantly, these bands were more pronounced in ethanolic and methanolic extracts than in their aqueous counterparts across both sample types. Several studies have reported the presence of these secondary metabolites in all three plants [21, 27, 28].

#### 2.3.2 Comparative HPLC Profiling of Plant Extracts From Wild and Hydroponic Sources

HPLC–diode array detector (DAD) fingerprints displayed apparent differences and similarities among the extracts from both sources, as shown in File S2, supporting information. For example, ethanolic and aqueous extracts of *C. asiatica* (Figure S2A,B) from both hydroponics and wild‐grown plants exhibited similar peaks at 10, 14, and 16 min. The water and ethanolic wild and hydroponic samples of *C. sumatrensis* (Figure S2C,D) exhibited comparable peaks at 10, 15, 48, and 50 min. All extracts from *J. betonica* exhibited prominent signals at 11, 47, 48, and 50 min in HPLC chromatograms, regardless of source and extraction solvent. The methanolic extracts from wild and hydroponic samples of this plant produced additional peaks at 30 and 38 min. In particular, *J. betonica* extracts from different growth conditions appear similar, with only minor variations in low‐abundance components (Figure S2E,F). Collectively, the extracts from wild sources consistently displayed more intricate chromatographic fingerprints, regardless of the solvent system used. The consistent peaks between wild and hydroponic samples represent core metabolites of each species, while the extra peaks in wild plant samples suggest stress‐related or soil‐derived compounds [29].

The detailed wild plant profiles suggest that hydroponic cultivation, though efficient, produces slightly different bioactive profiles. These differences underscore the need for fingerprint‐based quality control in medicinal plant extracts to ensure consistent therapeutic effects [30].

### 2.4 Comparative Volatile Chemical Composition of Wild vs. Hydroponic Raw Plant Samples

In this study, we used headspace GC–MS, in which volatile compounds with lower molecular weight are released into the headspace of a sample, and the gaseous phase is later injected into the chromatograph for separation and detection by a mass spectrometer. The volatile profiles of the three plants were identified and categorized into seven chemical classes: monoterpenes, sesquiterpenes, aldehydes, ketones, alcohols, hydrocarbons, and esters (Table S3A). The volatile organic compound (VOC) profiles of *C. asiatica*, *C. sumatrensis*, and *J. betonica* were similar, with some differences identified via a similarity search against the NIST14 mass spectral library. The percentage overlap and discrepancy of the three species were calculated using % Overlap = $\frac{S}{T}\times 100$, where *S* is the number of shared compounds, *W* is the number of compounds found only in wild, *H* is the number of compounds found only in hydroponics, and *T* is the total, that is, *S* + *W* + *H*. The values were recorded in Table 4 in which *C. asiatica* exhibited the highest similarity (92.86% overlap), indicating strong metabolic stability under hydroponic cultivation, *C. sumatrensis* (82.76% overlap) and *J. betonica* (85.71% overlap) showed moderate discrepancies, with unique compounds emerging in both cultivation systems particularly more hydroponic‐exclusive volatiles in *J. betonica* (see Table S3A).

**TABLE 4:** Number and percentage of shared and unique volatile compounds in wild and hydroponic samples.

Species	Wild	Hydroponics	Shared	Total	Overlap (%)
*Centella asiatica*	5	0	65	70	92.86
*Conyza sumatrensis*	7	3	48	58	82.76
*Justicia betonica*	1	5	36	42	85.71

**FIGURE 2:**
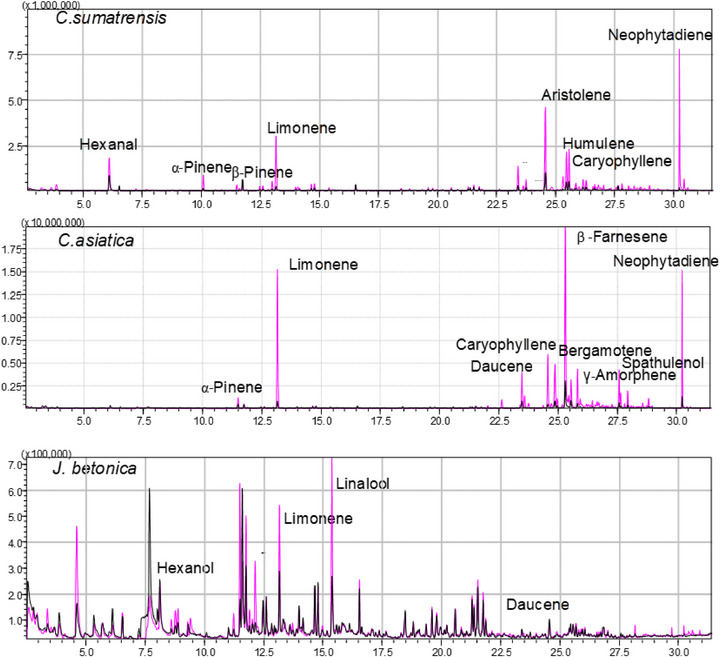
TIC chromatograms showing overlapping profiles of volatiles in wild (pink) and hydroponically grown (black) samples for the three plants. Chromatograms are shown for qualitative comparison; thus, no statistical test or *p* value applies. Colors indicate growth condition (pink = wild; black = hydroponic).

Many compounds were consistently found across all three species and growing systems, indicating essential metabolic processes. Similarities were observed in monoterpenes such as limonene, aldehydes (e.g., α‐methylbutanal, pentanal, and hexanal), as well as alcohols like *trans*‐3‐hexen‐1‐ol and 3,7,11‐trimethyl‐1‐dodecanol, as shown in Table S3A. Some components were only present in certain plants, for example, linalool (a monoterpene) was only detected in both samples of *J. betonica* (Figure 2), sesquiterpenes such as aristolene was present only in *C. sumatrensis* samples collected from the wild (Table S3A), whereas daucene was present in both *C. asiatica* and *J. betonica* samples, suggesting that certain volatile compounds are likely linked to both phylogenetic and ecological factors. In *C. asiatica*, specific VOCs, such as β‐farnesene, limonene, and neophytadiene, were detected in both samples, but with a higher intensity in the hydroponic samples (Figure 2). In contrast, the VOC profiles of *J. betonica* samples from hydroponics and wild collections look the same. Metabolite levels can be enhanced by the type of fertilizer applied in hydroponic systems. In this study, NPK was used, as it supplies nitrogen and phosphorus essential for terpene synthesis and for the production of ATP and nicotinamide adenine dinucleotide phosphate (NADPH), which are necessary for terpenoid synthesis [31].

Notably, all identified volatiles possess known bioactivities. For instance, limonene (a monoterpenoid) found in all three plants has been reported to exhibit antimicrobial properties and promote wound healing [32, 33]. Our findings align with those of Seel et al. [21], who noted that *C. sumatrensis* is abundant in monoterpene and sesquiterpene compounds, such as caryophyllene, and displays antibacterial activity.

### 2.5 Feature‐Based Molecular Networking and Liquid Chromatography–Mass Spectrometry of the Extracts

#### 2.5.1 Comparative Growth Metrics Between Wild and Hydroponically Cultivated Plants to Evaluate Adaptability to Hydroponic Conditions

**FIGURE 3:**
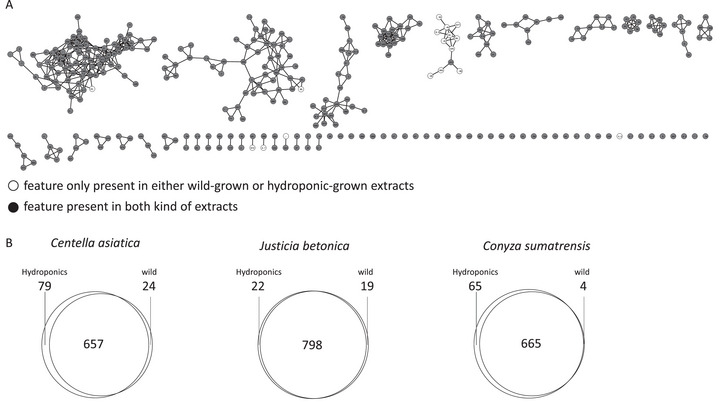
Feature‐based molecular networking (FBMN) of *Justicia betonica* plant extracts. (A) Molecular networks comparing features detected in wild‐grown and hydroponically grown extracts. Each node represents an MS/MS feature; filled circles indicate features present in both extract types, and open circles indicate features unique to either wild‐grown or hydroponic samples. (B) Venn style quantitative comparison of the number of features exclusive to hydroponic extracts, to wild‐grown extracts, and shared between them. All LC–MS/MS analyses were performed in positive ionization mode.

To assess the chemical composition of wild versus hydroponically cultivated plant extracts, feature‐based molecular networking (FBMN) was performed using liquid chromatography–mass spectrometry (LC–MS)/MS data acquired in positive ionization mode, as shown in the molecular network (Figure 3).

The network in Figure 3A, representing *J. betonica*, was selected as the representative network, with the others displayed in Figure S4. Most nodes are present in both wild and hydroponically grown plant extracts. The same pattern is observed for the other plants as well.

A quantitative comparison of feature counts (Figure 3B) revealed species‐specific responses to cultivation methods. In *C. asiatica*, both conditions shared 657 features, with 79 exclusive to hydroponic extracts and 24 to wild samples, suggesting greater metabolic diversity under hydroponic growth. *J. betonica* maintained a highly conserved metabolome across both conditions, with 798 shared features and few unique ones (22 in hydroponic, 19 in wild), indicating low responsiveness to the environment. Conversely, *C. sumatrensis* showed an increase in hydroponic‐specific features (65) compared to four unique to wild samples, supporting the idea that hydroponic systems can activate different metabolic pathways in this species. Similar trends were reported by Seel et al. [21], who identified a total of 270 metabolic features in both ethanol and aqueous extracts of *C. sumatrensis*. The FBMN results emphasize how molecular networking effectively visualizes chemical diversity and demonstrate that hydroponic cultivation is a valuable approach for modifying or enhancing secondary metabolite profiles in medicinal plants.

#### 2.5.2 Comparative LC–MS Metabolites Screening of Wild and Hydroponic Plant Extracts

LC–MS/MS analysis of *C. asiatica*, *J. betonica*, and *C. sumatrensis* extracts revealed unique yet overlapping phytochemical profiles, mainly flavonoids and triterpenoids, affected by cultivation method and solvent, as shown in File S3. The identification of compounds was performed based on retention times, accurate mass measurements, MS/MS fragmentation patterns, and molecular formula predictions validated through SIRIUS 6.2.2 and CSI:FingerID, applying stringent thresholds (SiriusScoreNormalized ≥ 95%, ZodiacScore ≥ 0.95, ConfidenceScoreApproximate ≥ 0.90) to ensure high‐confidence annotations.

In *C. asiatica*, we annotated four flavonoids and five terpenoids (see Table S3B), notably, both wild and hydroponic extracts exhibited the presence of quercetin‐3‐O‐glucuronoside (C_21_H_18_O_13_), identified at RT 4.95–4.96 min with experimental [M−H]^−^ at *m*/*z* 477 and [M−H]^+^ at *m*/*z* 478 with fragments at *m*/*z* 253, 301, and 325, detected in both ion modes. In addition, quercetin (C_15_H_10_O_7_) was predicted exclusively in wild ethanolic extracts at RT 5.16 min ([M−H]^−^, *m*/*z* 301, fragment at *m*/*z* 151). Kaempferol (C_15_H_10_O_6_) was consistently predicted across wild and hydroponic samples, appearing in both ion modes at RTs 5.70 and 9.70–9.77 min ([M+H]^+^, *m*/*z* 287; [M−H]^−^, *m*/*z* 285), with characteristic fragments at *m*/*z* 153, 285, and 287. Notably, hydroponic samples favored the accumulation of asiatic acid (C_30_H_48_O_5_), observed at RT 12.77–12.86 min with [M+Na]^+^ at *m*/*z* 511 and [M−H]^−^ at *m*/*z* 487. Conversely, wild ethanolic extract samples uniquely contained euscaphic acid (C_30_H_48_O_5_) at RT 7.99 min ([M+H]^+^, *m*/*z* 489) and medicagenic acid (C_30_H_46_O_6_) at RT 10.27 min ([M+H]^+^, *m*/*z* 503). Brahmic acid (C_30_H_48_O_6_) was also specific to hydroponics ethanol extracts, detected at RT 11.05–11.07 min ([M+NH_4_]^+^, *m*/*z* 522; [M−H]^−^, *m*/*z* 503). A further compound, kaempferol 7‐O‐glucoside (C_21_H_20_O_11_), was detected only in hydroponic ethanol extracts at RT 5.64 min ([M−H]^−^, *m*/*z* 447), consistent with its greater hydrophilicity. The common flavonoids of *C. asiatica*, such as quercetin and kaempferol, as well as terpenoids like asiatic acid and brahmic acid, have been extensively studied [34, 35]. Research shows that they possess biological activities, including antimicrobial and anti‐inflammatory effects [36].

For *J. betonica*, we were able to predict flavonoids (**3**), triterpenoids (**4**), and triterpene saponins (**1**) as shown in Table S3B. Thereby, a consistent presence of camelliaside A (C_33_H_40_O_20_) was found across wild and hydroponic samples in both extracts, with RT 3.82–3.88 min and detection in both ion modes ([M+H]^+^, *m*/*z* 757; [M+Na]^+^, *m*/*z* 779;[M−H]^−^, *m*/*z* 755), fragmenting to *m*/*z* 284, 285, and 287. Similarly, kaempferol‐3‐O‐rutinoside (C_27_H_30_O15) was detected in all conditions at RT 4.99–5.04 min ([M+H]^+^, *m*/*z* 595; [M+Na]^+^, *m*/*z* 617; [M−H]^−^, *m*/*z* 593), with characteristic fragments at *m*/*z* 284–287. In contrast, glaucaside C (C_42_H_68_O_14_), a triterpene saponin, appeared exclusively in wild methanol extracts at RT 8.75 min ([M+H]^+^, *m*/*z* 821) with fragments at *m*/*z* 437 and *m*/*z* 455. Katononic acid (C_30_H_46_O_3_) was annotated across both wild and hydroponic samples, appearing at RT 7.11–7.79 min ([M+H]^+^, *m*/*z* 471), with fragments at *m*/*z* 187–455. Pentacosanedioic acid (C_25_H_48_O_4_) was specific to wild extracts, detected at RT 21.99 min ([M−H]^−^, *m*/*z* 411). Moreover, quercetin 3‐(3R‐glucosylrutinoside) (C_33_H_40_O_21_) was found only in wild aqueous extracts at RT 3.32 min ([M+H]^+^, *m*/*z* 755; [M−H]^−^, *m*/*z* 753), while arjunglucoside II (C_36_H_58_O_10_), another triterpenoid glycoside, appeared exclusively in hydroponic samples at RT 10.92 min ([M−H]^−^, *m*/*z* 649). Queretaroic acid (C_30_H_48_O_4_) and oleanonic acid (C_30_H_46_O_3_), which are pentacyclic triterpenoids, were observed in hydroponic extracts at RTs 9.31 and 11.99 min with [M+H]^+^, *m*/*z* 473 and *m*/*z* 475, respectively. This is the first report of these flavonoids in *J. betonica*; however, kaempferol‐3‐O‐rutinoside has been found in *Justicia adhatoda* Linnaeus [37], whereas camelliaside A is primarily reported in the seeds of *Camellia sinensis* [38]. Triterpenoids, queretaroic acid (predicted for the first time), and oleanonic acid were found in both wild and hydroponic sample extracts. Oleanolic acid has also been reported in other *Justicia* species [39].

For *C. sumatrensis*, samples collected from the wild exhibited a rich array of both flavonoids (**6**) and prenol lipids (**8**). In contrast, the hydroponic counterparts had only prenol lipids as shown in Table S3C. Luteolin 7‐O‐diglucuronide (C_27_H_26_O_18_) was detected at RT 3.66 min ([M−H]^−^, *m*/*z* 639), with fragments at *m*/*z* 285 and 351, while chrysoeriol 7‐O‐diglucuronide (C_28_H_28_O_18_) was observed at RT 4.54 min ([M−H]^−^, *m*/*z* 653) with *m*/*z* 351 fragment. An unidentified flavonoid O‐glucuronide (C_23_H_16_O_12_) was noted at RT 4.95 min ([M−H]^−^, *m*/*z* 479) with fragment *m*/*z* 295, while acetylated luteolin 7‐O‐glucuronide (C_23_H_20_O_13_) and 4″‐O‐acetylglucuronide of kaempferol (C_23_H_20_O_13_) appeared at RT 5.52–5.93 min ([M−H]^−^, *m*/*z* 507) with fragments at *m*/*z* 285. In addition, a flavonoid 7‐O‐glucuronide (C_23_H_20_O_12_) was identified at RT 7.29 min ([M−H]^−^, *m*/*z* 491) with fragments at *m*/*z* 269. Unique to wild extracts was a triterpene saponin (C_37_H_60_O_11_) detected at RT 9.74 min ([M−H]^−^, *m*/*z* 677) and a diterpene glycoside (C_46_H_76_O_13_) at RT 22.56 min ([M−H]^−^, *m*/*z* 801) with fragments at *m*/*z* 277 and 293. Diterpenoids identified in wild samples included C_28_H_52_O_7_ at RT 22.87 min ([M−H]^−^, *m*/*z* 499), C_30_H_56_O_7_ at RT 23.81 min ([M−H]^−^, *m*/*z* 527), and C_23_H_46_O_3_ at RT 25.84 min ([M−H]^−^, *m*/*z* 387). In contrast, hydroponically grown *C. sumatrensis* yielded annotations belonging to primarily long‐chain fatty acids and diterpenoids, including 11,13‐dihydroxytetracos‐9‐enoic acid (C_24_H_46_O_4_) at RT 19.93 min ([M−H]^−^, *m*/*z* 413), octacosanedioic acid (C_28_H_54_O_4_) at RT 22.81 min ([M−H]^−^, *m*/*z* 483), and a diterpenoid (C_30_H_56_O_7_) at RT 23.85 min ([M−H]^−^, *m*/*z* 527). Our findings show triterpenoids in *C. asiatica*, conserved flavonoid and saponin families in *J. betonica*, and shared volatiles across cultivation methods, aligning with recent hydroponic research. A recent review article suggests that well‐managed soilless systems can maintain or enhance secondary metabolite levels compared to soil‐grown or wild‐grown plants, provided that nutrients, electrical conductivity (EC), and light are optimized, resulting in higher phenolic and terpenoid levels in medicinal plants [16]. Hydroponic studies on *C. asiatica* reveal high biomass and increased triterpene glycosides, such as asiaticoside and madecassoside, which enhance pharmaceutical processability [40]. LC–MS/MS profiling of *C. sumatrensis* confirmed the dominance of lipid‐like compounds, terpenoids, and flavonoid glucuronides, consistent with the findings of Seel et al. [21], despite variations in cultivation. Based on the above chemical relationships across species, we can demonstrate that hydroponics achieves phytochemical efficiency, including target‐class retention with reduced batch variability.

#### 2.5.3 Comparative Analytical Profiling of Wild and Hydroponically Cultivated Extracts

To thoroughly analyze the phytochemical profiles of *C. asiatica*, *C. sumatrensis*, and *J. betonica*, four complementary analytical techniques were employed (TLC, HPLC‐UV, HS‐GC–MS, and LC–MS/MS). Each method provided unique insights into the chemical composition and differences between wild and hydroponically grown samples. TLC enabled rapid qualitative screening of major metabolite classes. HPLC‐UV generated chromatographic fingerprints based on retention times, while HS‐GC–MS identified volatile compounds. LC–MS/MS provided detailed structural identification of secondary metabolites, including triterpenoids, flavonoid glycosides, and saponins. To summarize, these techniques demonstrate that hydroponic cultivation largely preserves the chemical diversity of wild plants, with only minor variations in metabolite levels across species. The main findings from each method are summarized in Table 5 below.

**TABLE 5:** Comparative summary of analytical methods and key phytochemical findings for wild and hydroponically cultivated plant extracts.

Method	Key parameters	Classes	Representative compounds (RT/*R*_f_)	Wild vs. hydroponic
TLC	Silica gel 60 F_254_; CHCl_3_:MeOH (8:2); UV 254/366 nm, anisaldehyde spray	Flavonoids, terpenoids, phenolics	*Centella asiatica*: *R*_f_ 0.20–0.84; *Conyza sumatrensis*: *R*_f_ 0.15–0.66; *Justicia betonica*: *R*_f_ 0.20–0.93	Wild extracts exhibited more bands while hydroponic samples had fewer, less intense spots
HPLC‐UV	C_18_ column; ACN/H_2_O (0.1% FA); *λ* = 220 nm; flow = 1 mL/min	Phenolics, triterpenoids	Major peaks: *C. asiatica* (14.2 min, asiatic acid); *C. sumatrensis* (12.5 min, terpenoid); *J. betonica* (18.7 min, flavonoid)	Core peaks are present in both, but wild extracts exhibited additional minor peaks, indicating a broader range of metabolites
HS–GC–MS	Shimadzu QP2010; Rxi‐5sil column; oven 35→300°C; EI 70 eV	Monoterpenes, sesquiterpenes	*C. asiatica*: limonene, β‐farnesene; *C. sumatrensis*: aristolene, caryophyllene oxide; *J. betonica*: linalool	High overlap (> 80%) between wild and hydroponic profiles; hydroponic samples showed higher abundance of key volatiles
LC–MS/MS	UHPLC–QTOF; C_18_ column; ACN/H_2_O + 0.1% FA; positive ESI	Flavonoids, triterpenoids	*C. asiatica*: asiatic and brahmic acids; *C. sumatrensis*: luteolin‐ and chrysoeriol‐glucuronides; *J. betonica*: kaempferol‐ and quercetin‐glycosides	Hydroponic *C. asiatica* enriched in asiatic/brahmic acids; wild *C. sumatrensis* richer in saponins; *J. betonica* largely consistent between sources

#### 2.5.4 Influence of Hydroponic Parameters on Phytochemical Composition

From a general perspective, controlled hydroponic nutrition regulated secondary metabolism without altering the qualitative chemotypes of the plants. For example, we observed that *C. asiatica* maintained stable triterpenoid profiles, *J. betonica* exhibited increased biosynthetic efficiency, and *C. sumatrensis* showed nitrogen‐responsive metabolic adjustments, as summarized in Table 6. It is evident that changes in the NPK ratios, specifically the 17–17–17 regimen in *C. asiatica*, could have supported consistent growth, with 230 g DW per plant, and resulted in a higher extract yield compared to wild plants (30.7% vs. 21.3%). LC–MS/MS analyses identified triterpenoids (asiatic acid and brahmic acid), flavonoids (quercetin‐3‐O‐glucuronoside and kaempferol‐7‐O‐glucoside) as the principal metabolites, in agreement with prior research on centelloside and flavonoid biosynthesis in *C. asiatica* under controlled nutrient conditions [41]. In *J. betonica*, delaying nutrient supplementation with NPK 17–17–17 until after 2 weeks improved both biomass (510 g DW per plant) and extract yield (16.2% compared to 8.9% in wild plants) with primary identified metabolites, including arjunglucoside II, katononic acid, oleanonic acid, camelliaside A, and kaempferol‐3‐O‐rutinoside. A proper nutrient balance promotes carbon flow into triterpenoid and flavonoid biosynthesis pathways, which accounts for the enhanced antibacterial and anti‐inflammatory properties observed [42]. Growing *C. sumatrensis* with nitrogen‐rich NPK 19–19–19 resulted in the highest biomass, reaching up to 700 g DW per plant, but yielded less extract (13.4% ethanolic and 6.2% aqueous). The plant's chemical profile was primarily composed of prenol lipids and flavonoid glucuronides, such as luteolin 7‐O‐diglucuronide and chrysoeriol 7‐O‐diglucuronide, reflecting nitrogen‐driven shifts toward terpenoid biosynthesis [31]. This demonstrates a nutrient‐driven carbon–nitrogen trade‐off, where high nitrogen levels enhance growth but reduce flavonoid diversity [43]. These findings support the use of hydroponic systems for the sustainable production of medicinal phytochemicals through optimized nutrient management.

**TABLE 6:** Presents the correlations between cultivation parameters and plant properties for hydroponically grown medicinal plants.

Species	NPK regime and schedule	Growth (days)	Fresh:dry biomass per plant (g)	Dominant compounds	Extraction yield (% w/w) (ethanolic:aqueous)	Proposed hydroponic–chemical relationship
*Centella asiatica*	NPK 17–17–17 (0.5 g/L at transplanting; fortnightly supplementation; monthly renewal)	90	1700:230	Asiatic acid, brahmic acid, quercetin‐3‐O‐glucuronoside, kaempferol‐7‐O‐glucoside, and monoterpenes (limonene, β‐farnesene)	30.7:9.6	Continuous nutrient supply maintained high triterpenoid content (asiatic /brahmic acids) and preserved flavonoid diversity comparable to wild plants
*Justicia betonica*	NPK 17–17–17 (0.5 g/L introduced after 2 weeks; monthly renewal)	90	2800:510	Camelliaside A, kaempferol‐3‐O‐rutinoside, arjunglucoside II, oleanonic acid, katononic acid	16.2:9.6	Balanced nutrient regime favored the biosynthesis of triterpenoids (arjunglucoside II, katononic acid) and flavonoid glycosides, thereby enhancing the overall yield compared to wild plants
*Conyza sumatrensis*	NPK 19–19–19 (1 g/L at transplanting; repeated 2 days; monthly renewal)	90	4200:700	Prenol lipids, luteolin 7‐O‐diglucuronide, chrysoeriol 7‐O‐diglucuronide, acetylated luteolin 7‐O‐glucuronide	13.4:6.2	Elevated nitrogen promoted lipid‐derived terpenoid production while moderately reducing flavonoid diversity, reflecting nutrient‐driven metabolic adjustment.

*Note*: It links nutrient regimes, growth duration, biomass, and dominant phytochemicals to extraction yields. The values summarize cultivation conditions and results for *C. asiatica*, *J. betonica*, and *C. sumatrensis* grown under standardized hydroponic conditions. Extraction yields are based on single determinations (see Table 2) conducted with the same solvent ratios and extraction durations.

### 2.6 Comparative Antibacterial Screening of the Three Medicinal Plants

Antimicrobial activity is crucial for effective medicinal products used in wound healing. We thus subjected the different extracts of *C. asiatica*, *J. betonica*, and *C. sumatrensis* from the two cultivation conditions to susceptibility tests against Gram‐positive and Gram‐negative bacterial strains [44] (Table 7). In our findings, extracts from *C. asiatica* from both sources showed inhibition zones against *Pseudomonas fluorescens*; in contrast, only CAEE showed activity against *Escherichia coli*, *Bacillus subtilis*, and *P. fluorescens*. This can be attributed to ethanol's ability to extract more phytochemicals with antimicrobial activity than water [45]. Many studies have reported that ethanolic extracts of *C. asiatica* exhibit potent antibacterial activity across various extraction methods [44]. The presence of monoterpenes such as limonene, α‐pinene, and γ‐terpinene (as shown in Table S3A) may have contributed to the antibacterial potential of *C. asiatica*. In addition, a study reported the presence of these components in the oils of *C. asiatica* and suggested that they could be responsible for the activity [46]. Moreover, the antibacterial activity of the ethanolic extracts of *C. asiatica* may be linked to components like asiatic acid, brahmic acid, and other triterpenoids identified via LC–MS/MS analysis (Table S3B). These compounds are known to damage bacterial membranes, disrupt ion transport, and interfere with energy production, resulting in cell leakage and ultimately cell death [47, 48].

**TABLE 7:** shows the antibacterial activity of aqueous, ethanolic, and methanolic extracts of *Centella asiatica*, *Justicia betonica*, and *Conyza sumatrensis* from wild and hydroponic sources at 100 mg/mL.

		*C. asiatica*				*J. betonica*				*C. sumatrensis*			
Extraction solvent	Cultivation method	*Escherichia coli*	*Bacillus subtilis*	*Pseudomonas fluorescens*	*Saccharomyces cerevisiae*	*E. coli*	*B. subtilis*	*P. fluorescens*	*S. cerevisiae*	*E. coli*	*B. subtilis*	*P. fluorescens*	*S. cerevisiae*
Ethanolic/methanolic	Wild	1.0	0.6	1.0	0.9	0.9	0.0	0.0	0.0	0.7	0.9	0.9	0.9
	Hydroponics	0.8	0.7	0.6	1.0	0.8	0.0	0.0	0.7	0.9	0.9	0.9	1.0
Aqueous	Wild	0.0	0.0	0.6	0.0	0.0	0.0	0.9	0.0	0.0	0.0	0.0	0.0
	Hydroponics	0.0	0.0	0.9	0.0	0.0	0.0	0.8	0.0	0.0	0.0	0.8	0.0
Positive control		3.0	2.4	1.9	1.5	2.9	2.4	2.0	1.5	2.9	2.4	2.1	1.9

*Note*: Ethanol extracts of *C. sumatrensis* had the strongest inhibition, with zones of 0.9–1.0 cm against *E. coli*, *B. subtilis*, and *P. fluorescens*. *C. asiatica*'s ethanolic extract inhibited *E. coli* and *P. fluorescens* with a zone of 1.0 cm, while *J. betonica*'s methanolic extracts showed zones between 0.7 and 0.9 cm. Aqueous extracts had weak or no inhibition, zones less than 0.6 cm. Positive controls were gentamycin (3.0 cm) and amphotericin B (1.9 cm). Data are single measurements for comparison.

The *J. betonica* methanol extracts from both cultivation techniques showed modest antimicrobial activity against *E. coli* but did not affect *B. subtilis* and *P. fluorescens*. Conversely, the aqueous extracts both inhibited *P. fluorescens*, indicating the presence of specific hydrophilic antibacterial compounds targeting this bacterium. Notably, the methanolic extract from the hydroponic plants inhibited *Saccharomyces cerevisiae*, a result not observed in the wild sample. A study in India tested aqueous and methanol extracts of *J. betonica* against various bacterial strains, revealing potential antibacterial effects attributed to flavonoids, terpenoids, and alkaloids [22]. Pentacyclic triterpenoids, including katononic acid and oleanonic acid, which were tentatively identified in *J. betonica* extracts, might have played a role in the notable antibacterial activity observed. Research on these isolated compounds showed effectiveness against both Gram‐negative and Gram‐positive bacteria [49, 50].

The antibacterial activity of CSEE was notably high. Both wild and hydroponic samples showed prominent inhibition zones against *E.coli*, *B. subtilis*, *P. fluorescens*, and *S. cerevisiae*, confirming effective antimicrobial properties regardless of cultivation method. In contrast, aqueous extracts from both wild and hydroponic sources showed no detectable antibacterial activity, indicating that the main antimicrobial compounds in this species are primarily soluble in ethanol. A recent study by Seel et al. [21] found that ethanolic leaf extracts of *C. sumatrensis* exhibited higher antibacterial activity (MIC = 0.313 mg/mL) compared to aqueous extracts. Interestingly, all *C. sumatrensis* samples (see Table S3A) contained sesquiterpenes, specifically caryophyllene oxide [51], and flavonoids such as chrysoeriol‐7‐O‐diglucuronide [52], both of which have been previously recognized for their antibacterial effects.

While these plant extracts are not as potent as standard antibiotics like gentamycin sulfate or amphotericin B, their moderate activity, particularly against *E. coli* and *P. fluorescens*, warrants further investigation. This supports the traditional use of these plants for treating infected wounds and emphasizes hydroponics as an eco‐friendly cultivation method that preserves bioactivity. The antibacterial activity of these extracts may be partly due to monoterpenes such as limonene, present in both wild and hydroponic samples (Table 7), and to bioactive metabolites including flavonoids and terpenoids (Table S3B), all of which are well documented for their antimicrobial potential [53, 54]. While the antibacterial findings could not be replicated statistically, similar inhibition patterns appeared regardless of species or extraction solvent, which lends support to broader comparative conclusions.

### 2.7 Comparative Anti‐Inflammatory Activity and Cytotoxicity Effect of the Three Plants

#### 2.7.1 In Vitro Anti‐Inflammatory Activity of Plant Extracts Based on IL‐6 and IL‐8 Modulation

Wound healing involves four interconnected stages: inflammation, neovascularization, re‐epithelialization, and matrix remodeling, all of which are influenced by mediators such as interleukins [1]. In our study, we aimed to investigate how extracts from the selected plants affect interleukin secretion. Thereby, we focused on the initial stage (inflammatory response), where IL‐6 and IL‐8 play a crucial role in recruiting immune cells and keratinocytes. We assessed the release of IL‐6 and IL‐8 from human keratinocytes in a concentration and plant extract‐dependent manner following TNF‐α‐induced inflammation, using a sensitive and specific antibody‐based ELISA method that quantifies cytokines without the need for sample purification, as shown in Figure S5A–F.

In our assays, we observed a significant reduction in IL‐6 release only with aqueous extracts of *C. asiatica* and *C. sumatrensis* (100 µg/mL). In the case of *C. asiatica*, the anti‐inflammatory effects may be linked to flavonoids and diterpenoids, which were predicted to be hydroponic, as shown in Table S3B. Aqueous extraction (especially hot‐water decoction) likely concentrates more polar compounds, such as flavonoid glycosides, which could support the observed decrease in IL‐6 secretion in *C. asiatica* and *C. sumatrensis*. In addition, *C. asiatica* extracts specifically contained key flavonoids (see Table S3B), such as kaempferol and quercetin, which have been known to exhibit potent anti‐inflammatory effects [55]. The significant reduction in IL‐6 release observed with CAAEs is consistent with previous in vivo evidence demonstrating attenuation of inflammation and apoptosis, accompanied by lowered IL‐6, TNF‐α, and other proinflammatory markers [56]. In the case of *C. sumatrensis*, this effect was, interestingly, absent at higher concentrations. The species in the genus *Conyza* have demonstrated to possess anti‐inflammatory activities [57], for example, a study on *Conyza bonariensis* through inhibition of nitric oxide production mechanism showed a decrease in IL‐6 secretion at 100 µg/mL [58]. The increase in IL‐6 secretion for JBMEs from the wild (500 µg/mL) highlights the need for caution when considering this extract for topical applications aimed at reducing inflammation, this is the first report on *J. betonica*; however, a study of the anti‐inflammatory potential of procumbenoside B isolated from *Justicia spicigera*, a medicinally used plant in Mexico from genus *Justicia* revealed similar results [59]. In the case of IL‐8, none of the plant extracts evaluated (from hydroponics or wild‐type), nor the solvent or its concentration, exhibited any statistically significant modulation of cytokine release compared to the TNF‐α‐stimulated controls. This suggests a selective cytokine‐modulation profile for these plant extracts, with a specific impact on IL‐6 rather than on IL‐8 in this in vitro anti‐inflammatory model. However, more detailed studies have to corroborate these observations.

#### 2.7.2 Cytotoxicity Assessment of Plant Extracts Using MTT Assay

After the induced inflammatory response, we conducted a viability assay to examine the cytotoxic effects of the plant extracts on the cells and ensure their viability throughout the experiment. This assay relies on the mitochondrial conversion of dye, which is directly associated with cellular metabolic activity. The assay is advantageous due to its practicality, speed, reproducibility, and cost‐effectiveness. Our results indicated that all extracts (*C. sumatrensis*, *C. sumatrensis*, and *J. betonica*) exhibited no significant cytotoxicity and maintained cell viability similar to the untreated control group (Figure S5G–I), with over 95% cell viability at concentrations up to 500 µg/mL. Based on published cytotoxicity classification criteria for natural extracts, materials with ≥ 75% viability at 500–1000 µg/mL are considered low in toxicity. This suggests that the tested plant extracts are safe for wound‐healing applications [60, 61]. These findings confirm that the plant extracts are noncytotoxic in our assay and that the observed differences in cytokine release are not due to cellular damage or metabolic inhibition. The absence of cytotoxicity observed for *C. asiatica* extracts in the present study is consistent with the findings of Bikiaris et al. [62], who reported that CAEEs did not exhibit cytotoxic effects. To the best of our knowledge, this is the first report demonstrating the noncytotoxic nature of *C. sumatrensis* and *J. betonica* extracts under comparable experimental conditions.

### 2.8 Sustainability and Environmental Impact: A Life Cycle Perspective

Transitioning from wild harvesting to hydroponic cultivation provides sustainability benefits. Collecting medicinal plants from nature is traditional and cultural, but it harms ecosystems, causing species decline and habitat loss [63]. Hydroponic methods offer a sustainable alternative, reducing the environmental impacts of supply chains.

A recent study shows hydroponic methods outperform traditional soil farming in water efficiency, land use, and some emissions. When comparing aeroponic, hydroponic, and soil cultivation of plants like *Coffea arabica* and *Senecio bicolor*, it was found that soil systems used less electricity, whereas hydroponic and aeroponic systems saved more water and produced higher biomass. The main environmental impacts of hydroponic systems are electricity use and nutrient inputs [64]. In a life cycle assessment of lettuce, Banboukian et al. [65] observed that hydroponic farming uses less water than open‐field and greenhouse methods with recirculation. However, it demands more energy for lighting and climate management, resulting in a larger carbon footprint unless renewable energy sources are employed.

Therefore, in hydroponic cultivation, despite higher initial costs for materials, infrastructure, and energy, it can be more environmentally sustainable over multiple harvests if systems use low‐carbon energy and recycle nutrients efficiently. In such cases, its impact per unit biomass or active compound is more advantageous than wild harvesting, which can cause habitat destruction, result in waste, and require long‐distance transport.

## 3 Materials and Methods

### 3.1 Selection, Authentication and Mini‐Ethnobotanical Survey of the Medicinal Plants

#### 3.1.1 Literature‐Guided Selection of Traditional Medicinal Plants for Wound Healing

A literature search was conducted through online resources on medicinal plants used in wound healing, utilizing public databases such as PubMed, ScienceDirect, Scopus, and Google Scholar. The search adhered to straightforward criteria for selecting candidate plants, including well‐documented ethnomedicinal uses for wound healing, reported phytochemical composition, and pharmacological activities such as antimicrobial or anti‐inflammatory properties. The feasibility of cultivating plants in a greenhouse hydroponics system was part of the screening criteria, which included assessing whether the plants could develop a taproot during the process. Thus, after thorough screening, the review identified three plants: *C. sumatrensis*, *J. betonica*, and *C. asiatica*.

#### 3.1.2 Taxonomical Authentication of the Selected Medicinal Plants

The three selected medicinal plants were collected from Namagunga village, Lugazi Diocese, Buikwe District, and voucher specimens were verified by a botanist (Clement Olusoji Ajayi) at the Department of Pharmacy, Faculty of Medicine, Mbarara University of Science and Technology, on April 3, 2023. The aerial parts of *C. sumatrensis* (Retz.) E. Walker, *J. betonica* L., and *C. asiatica* (L.) Urb. were prepared and assigned the collection numbers IK‐2023‐001, IK‐2023‐002, and IK‐2023‐003, respectively. Their scientific names were checked against the International Plant Name Index (www.ipni.org) and the Kew Royal Botanic Garden (www.theplantlist.org) online databases, which include flora from East Africa and Africa, accessed on April 4, 2023.

#### 3.1.3 Ethnobotanical Field Study of *C. sumatrensis* (Retz.) E. Walker, *J. betonica* L., and *C. asiatica* (L.) Urb. in Lugazi Diocese, Buikwe District, Uganda

**FIGURE 4:**
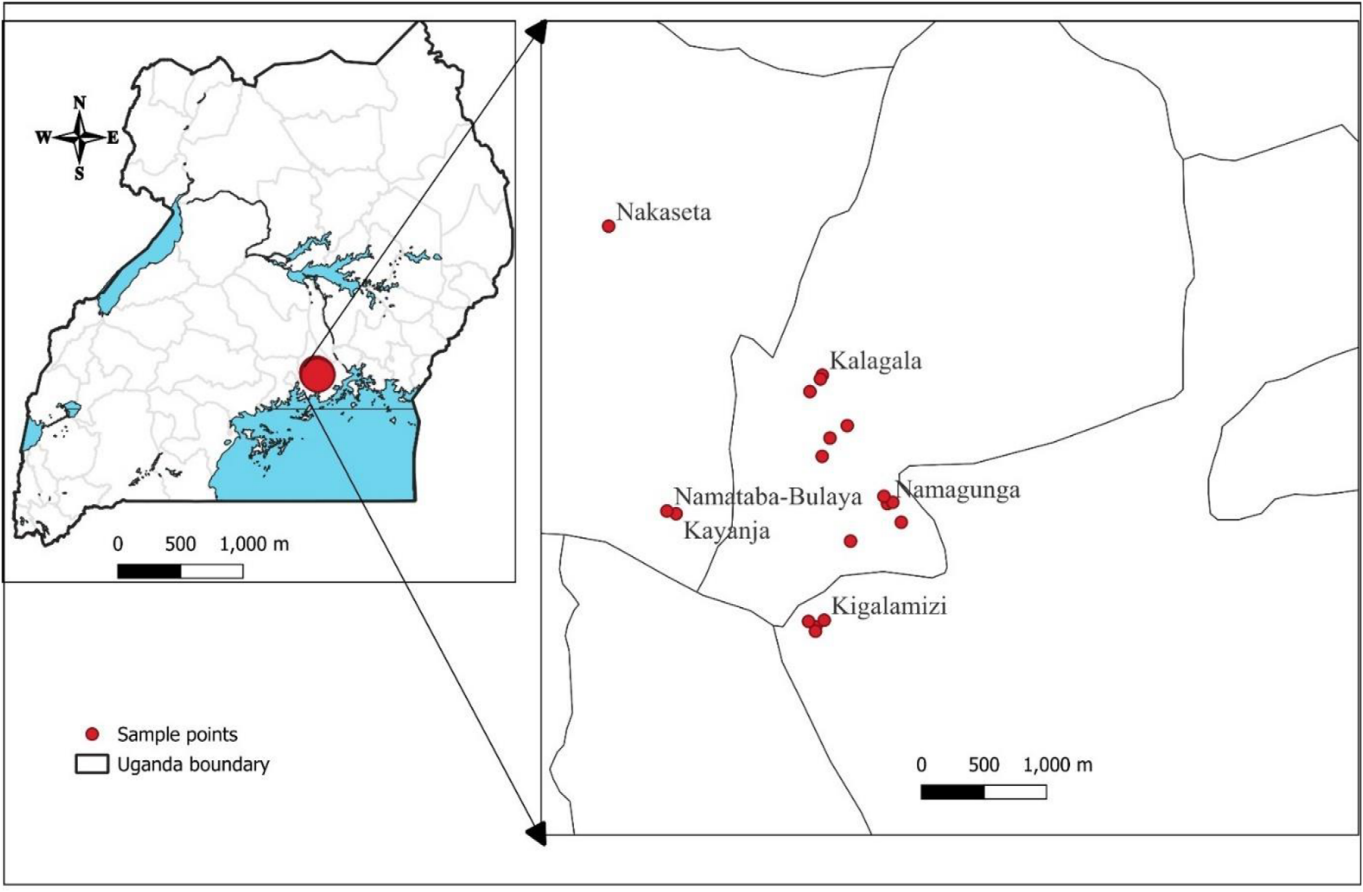
Visited villages in Lugazi Diocese, Buikwe District (with a thick red dot), Uganda.

A literature‐guided ethnobotanical survey was conducted to confirm the traditional use and local availability of three medicinal plants in villages selected through purposive sampling in the Lugazi Diocese, Buikwe District, Uganda (Figure 4). This region has a rich biodiversity and a long history of traditional plant use [20]. Before the survey, informed consent was sought from the selected participants. The survey focused on distinguished community members with reputable knowledge of medicinal plants in wound healing. The study involved semi‐structured interviews with herbalists, traditional healers, and elderly individuals. The data from this survey were analyzed using descriptive statistics.

### 3.2 Hydroponic Cultivation of Selected Medicinal Plants

#### 3.2.1 Greenhouse Site Description

**FIGURE 5:**
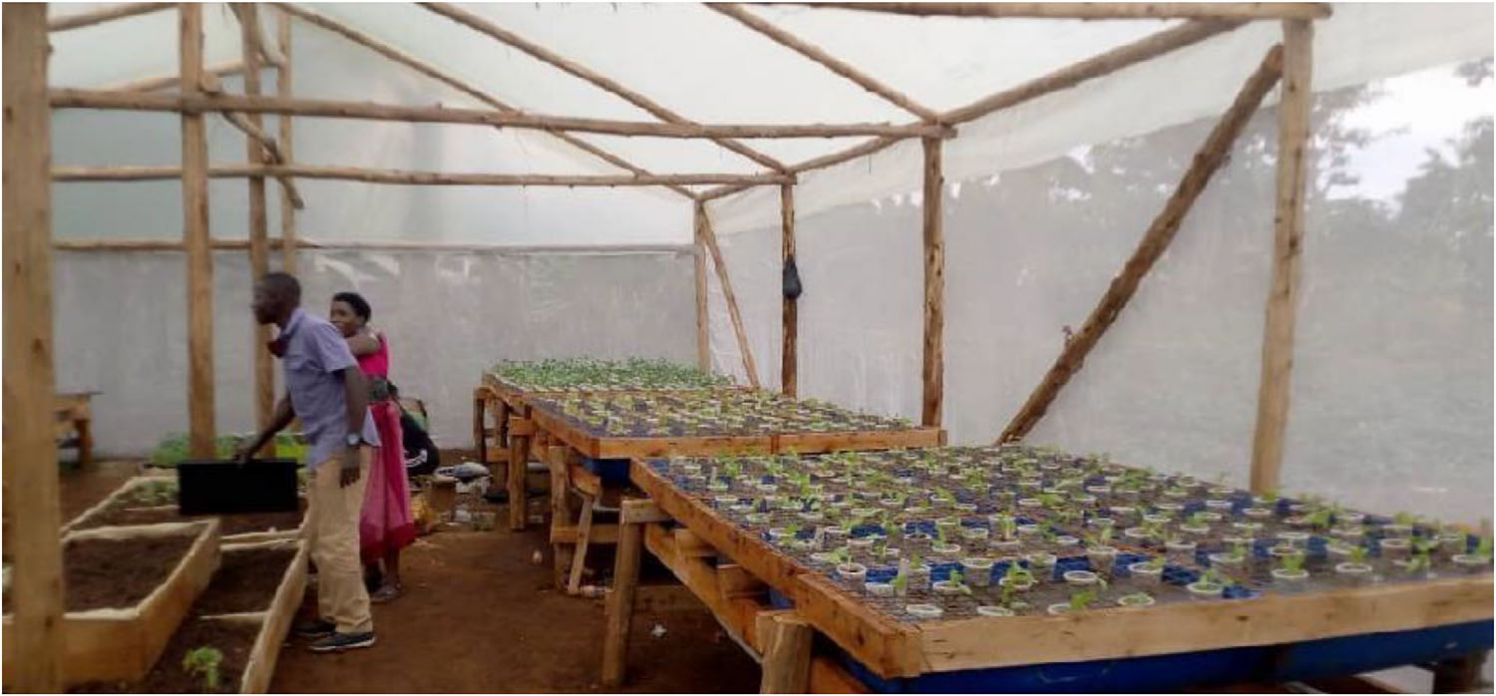
Greenhouse hydroponics system in Namagunga, Lugazi Diocese, Buikwe District.

The greenhouse hydroponic system used in this study was established in Namagunga, Lugazi Diocese, Buikwe District, Uganda (Figure 5). It was oriented north‐south to align with the prevailing east‐west wind patterns, enhancing structural stability against fierce winds [66]. The structure was built in an open area, free from light obstructions, ensuring consistent light exposure essential for plant growth. For durability, the greenhouse framework utilized locally sourced wooden poles treated with “IMIDA,” an anti‐termite chemical. A UV‐blocking polyethene sheet covered the structure, allowing the best light transmission while minimizing the effects of harmful UV rays on plants [67]. Temperature regulation was achieved through side vents equipped with shade nets, which improved airflow and reduced heat buildup. In addition, these vents served as pest barriers, trapping insects that attempted to enter the greenhouse.

#### 3.2.2 Hydroponic System, Growth Conditions, Planting, and Cultivation Protocols

The hydroponic system was constructed with grow beds using halved plastic drums supported by wooden frames and lined with wire mesh [68]. To aid efficient water drainage and recycling of nutrients, polypropylene (PP‐R) pipes were installed [69]. Each medicinal plant species required a different nutrient composition for growth at a pH of 7. *C. sumatrensis* received an initial NPK 19:19:19 solution (1 g/L) during the initial planting phase and was applied twice consecutively. *J. betonica* was grown initially in fresh water, and after 2 weeks, the same fertilizer in liquid form but with a different ratio (NPK 17:17:17, 0.5 g/L) was introduced. For *C. asiatica*, a similar regimen to that for *J. betonica* was used biweekly, with regular topping up of water to maintain temperature and oxygenation, thereby preventing root rot and nutrient depletion. Propagation methods varied according to species requirements. *C. asiatica* was propagated using vines and suckers, while *J. betonica* was established from vine cuttings or runners emerging from the central stem. *C. sumatrensis* was sown from seeds. Cuttings or seeds were placed in grow beds for each species and maintained at shallow water levels to promote optimal growth. The medicinal plants were closely monitored, particularly during hot seasons, to ensure their optimal growth. Other key factors for reproducibility involved maintaining a temperature of 20°C–25°C, a relative humidity of 60%–70%, and a consistent 12‐h light/12‐h dark photoperiod to minimize variation in plant biomass and metabolite accumulation, keeping inter‐batch differences within ±5%.

### 3.3 Harvesting and Extraction of Wild‐Type and Hydroponically Grown Medicinal Plants

The leaf plant materials collected from one of the surveyed locations and those from hydroponic cultures were dried in the shade for 2 weeks at outside temperatures (20°C–25°C). After drying, they were crushed into coarse size using an electric lab grinder and later extracted by decoction or maceration; these methods were adopted from the ethnobotanical survey as reported by the respondents. For decoction, 35 g of the powdered leaf material was mixed with 350 mL of distilled water in a 500 mL conical flask, sealed with aluminum foil, and heated at 100°C for 15 min. After cooling, the mixtures were filtered using Whatman filter No. 1, which was followed by lyophilization using a freeze‐dryer. The final dried extracts were weighed and stored at 4°C for analysis. All three plants underwent maceration; specifically, 35 g of *J. betonica* leaves were macerated with 350 mL of 99.5% analytical‐grade methanol, while *C. sumatrensis* and *C. asiatica* were each extracted with 350 mL of 70% analytical grade ethanol and covered with aluminum foil for 48 h with regular shaking every 6 h. The specific medicinal plant guided the selection of organic extraction solvents, determined through a literature review on phytochemicals with potential wound‐healing properties commonly used in the extraction of each chosen plant [70]. The extracts were filtered and concentrated using a rotary evaporator (Heidolph Hei‐VAP Core rotary evaporator; Heidolph Instruments, Schwabach, Germany) coupled to a Ecodyst EcoChyll S cooler (Ecodyst, Apex, NC, USA) under specific conditions; that is, for methanol extracts, the pressure was maintained at 45 mbar and 45°C, and for ethanol extracts, at 50 mbar and 35°C at a rotation speed of 150 rpm for all. Extraction yields were determined from single representative extractions conducted under standardized conditions, due to the limited availability of hydroponic biomass; therefore, statistical replicates were not included. The dried extracts were weighed and stored at 4°C for further use.

### 3.4 TLC Profiling of the Extracts

TLC profiles of the plant extracts were performed to compare samples from wild and hydroponic cultivations following a procedure by Seel et al. [21]. Spotting was performed by applying 10 µL of each extract (1 mg/mL) to silica gel 60 F254 TLC plates (Macherey‐Nagel, Germany). The plates were developed using a chloroform:methanol (8:2, v/v) mobile phase; initial detection was conducted under visible light and later visualized under short‐wavelength UV (254 nm) and long‐wavelength UV (366 nm). To enhance the detection of specific phytochemical groups such as flavonoids, terpenoids, and phenolic compounds, we derivatized the dried plates with *p*‐anisaldehyde sulfuric acid reagent, then dried at 105°C in an oven, and subsequently examined for the presence of targeted compounds through color analysis of spots, with their *R*_f_ values calculated.

### 3.5 HPLC–DAD Phytochemical Profiles Fingerprinting of Extracts

For the HPLC analysis of plant extracts (20 mg/mL in 5% acetonitrile) a Hitachi Primade HPLC system coupled with a 1430 DAD was used. This setup included a 1210 autosampler, an 1110 pump, and a 1310 column oven. Chromatographic separations were conducted on a Nucleodur 100–5 C_18_ EC column with a length of 250 mm, an internal diameter of 4 mm, a particle size of 5 µm, and a pore size of 110 Å (Macherey‐Nagel). Acetonitrile (100%) served as Solvent A, while a mixture of water and acetonitrile (95:5 v/v) was used as Solvent B, following this gradient: from 0.0 to 40.0 min, 95% B; from 40.0 to 45.0 min, 80% B; from 45.0 to 50.0 min, back to 95% B; and from 50.0 to 51.0 min, remaining at 95% B. The flow rate was set to 0.5 mL/min, with DAD acquisition performed over a wavelength range of 190–900 nm and specifically monitoring at 220 nm. Chromatograms were captured at 205 nm during a 60‐min run to identify potential fingerprints. The column chamber temperature was kept at 30°C, and the injection volume was 2.0 µL. Data processing utilized Primade software.

### 3.6 Headspace GC–MS Analysis of Volatile Components of the Raw Plant Materials

GC–MS headspace analysis was conducted using the method described by Seel et al. [21]. Fifty milligrams of powdered leaf material from the three plants was briefly placed in a 20 mL headspace vial, which was then tightly sealed, and the vial was equilibrated at 70°C for 8 min. A 1 mL part of the volatile headspace was extracted under 50 kPa pressure and passed through a Tenax trap at 5°C. The trapped volatiles were thermally desorbed at 300°C and introduced into the GC–MS system in split mode (5:1). Chromatographic separation was performed on a Shimadzu QP2010 GC–MS, using helium as the carrier gas at a flow rate of 0.95 mL/min. The oven temperature was initially set to 35°C for 2 min, then increased to 150°C at 5°C/min, and finally to 300°C at 15°C/min. Mass spectra were obtained through electron ionization (70 eV) at a source temperature of 230°C. The resultant data were analyzed using GCMSsolution v4.20 software, and chemical compounds were tentatively identified by comparison with the NIST14 spectral library. The relative peak areas were calculated as percentages of the total ion current (TIC).

### 3.7 FBMN and LC–MS/MS Analysis of the Extracts

#### 3.7.1 FBMN

The chemical relationships among the detected features were explored by processing LC–MS/MS data using MZmine (v4.0) and submitting it to the Global Natural Product Social Molecular Networking (GNPS) platform for FBMN analysis, following the workflow protocol described by Seel et al. [21]. Raw data were centroided, converted to mzML format, then aligned and filtered for peak quality and presence in biological replicates. The GNPS FBMN workflow generated a molecular network with a cosine similarity threshold of 0.6 and a minimum of four matched fragments per edge. Putative structural annotations were obtained via library searches against GNPS and MassBank databases. Compound classification was supported by using *insilico* tools, including SIRIUS (v5.8.5), CANOPUS, and ClassyFire. Network visualization was performed in Cytoscape (v3.10.2).

#### 3.7.2 LC/MS/MS Analysis of the Extracts

Untargeted LC–MS analysis was conducted to profile semi‐polar constituents in the extracts, following the detailed protocol described by Seel et al. [21]. Analyses were conducted using a UHPLC system (Ultimate 3000, Thermo Fisher Scientific) coupled to a high‐resolution QTOF mass spectrometer (Impact II, Bruker Daltonics), operated in positive ionization mode with data‐dependent MS/MS acquisition. Approximately 5 µL of the sample extracts for each plant were injected and separated on a C_18_ column (100 × 3 mm, 5 µm particle size) using a gradient of acetonitrile and 0.1% formic acid in water at 0.6 mL/min. The gradient progressed from 15% to 100% acetonitrile over 22 min and was held for an additional 12 min. The capillary voltage was set to 3700 V, with a scan range of *m*/*z* 100–1000. The acquired chromatograms were visualized using Compass DataAnalysis 4.2 software (Bruker).

#### 3.7.3 Metabolite Prediction, Identification, and Annotation

The UHPLC–QTOF data were converted to mzML format using the peak‐picking algorithm in MSConvert, version 3.0.25190‐0392f93, from ProteoWizard (https://proteowizard.sourceforge.io/download.html). The converted files were then uploaded to the Windows version of SIRIUS+CSI:FingerID GUI and CLI—Version 6.2.2 (2025‐06‐03) (https://bio.informatik.uni‐jena.de/software/sirius/). Metabolite identification was performed using MS/MS data in both positive and negative modes. The molecular formula identifier (MFI) employed a De novo up + up bottom strategy targeting *m*/*z* below 400, allowing elements like H, C, N, O, and P, with auto‐detection of other elements. For fingerprint prediction, the CSI:FingerID method was used, with PubChem as a fallback in structure database searches. The compound class predictor (CANOPUS) within SIRIUS software was activated, with a mass accuracy threshold of ≤ 10 ppm. De novo structure generation utilized the MSNovelist tool within SIRIUS. Spectral library searches were carried out with a precursor deviation threshold of  ≤ 20 ppm for both wild and hydroponically cultivated extracts, using CSI:FingerID across 23 libraries—such as Biocyc, CHEBI, COCONUT, FooDB, GNPS, HMDB, HSDB, KEGG, KNApSAcK, LOTUS, LipidMaps, Maconda, MeSH, MiMeDB, NORMAN, Plantcyc, PubChem bio & metabolites, PubChem drug, PubChem food, PubChem safety & toxic, SuperNatural, TeroMOL, and YMDB [71]. Candidate molecular formulas were first predicted by SIRIUS based on isotope patterns and fragmentation trees and ranked using the SiriusScoreNormalized. High‐scoring formulas (typically > 95%) were retained. To refine formula confidence, ZODIAC was applied to re‐rank formulas using a probabilistic network model. Only formulas with ZodiacScore ≥ 0.95 were considered high confidence. For each retained formula, CSI:FingerID predicted molecular fingerprints and searched structural databases (e.g., PubChem). Candidate structures were ranked by CSI:FingerIDScore, which is a relative score. To assess structural reliability, the ConfidenceScoreApproximate (COSMIC) was used. This machine learning‐derived score (0–1) estimates the likelihood that the top‐ranked structure is correct; scores of 0.95 or higher are considered high confidence.

### 3.8 Pharmacological Activities of the Medicinal Plant Extracts

#### 3.8.1 Antibacterial Sensitivity Test

The antimicrobial activity was conducted using *E. coli* (DSM11250), *B. subtilis* (ATCC 6051 or DSM10), and *P. fluorescens* (WS 1760 or DSM 6147), provided by Leibniz Institute DSMZ‐German Collection of Microorganisms and Cell Cultures GmbH. *S. cerevisiae* (baker's yeast) was bought from local grocery stores (Leipzig, Germany). The antibacterial sensitivity assay was conducted using the disc diffusion assay with commonly used antibiotics [21]. Nutrient agar was employed for *B. subtilis* and *P. fluorescens*, Luria–Bertani (LB) agar for *E. coli*, and Universal medium for *S. cerevisiae*. All media were prepared according to the guidelines of the Deutsche Sammlung von Mikroorganismen und Zellkulturen (DSMZ). Each microorganism was subcultured overnight in its respective liquid medium at the appropriate temperature. Following incubation, 200 µL of each bacterial culture was evenly spread on the corresponding solid media. Discs measuring 6 mm in diameter, impregnated with 10 µL of 100 mg/mL for each extract and a positive control antibiotic (gentamycin sulfate, 5 µL), were placed on the agar surface. The plates were incubated at 37°C for *E. coli* and at 30°C for the other bacterial strains for 24 h. After incubation, the inhibition zones around the discs were measured to assess and interpret antibiotic sensitivity. The inhibition zones were measured once per treatment as a preliminary screening to compare relative antibacterial trends between wild and hydroponic samples. No statistical inference was applied.

#### 3.8.2 In Vitro Anti‐Inflammatory and Cytotoxicity Assays

##### 3.8.2.1 Cell Culture Maintenance

Human immortalized keratinocytes (HaCaT cells) (RRID: CVCL_0038) obtained from CLS Cell Lines Service (Eppelheim, Germany) were cultured in Dulbecco's modified Eagle medium (DMEM) supplemented with 10% fetal bovine serum (FBS) and 1% penicillin–streptomycin at 37°C in a humidified atmosphere containing 5% CO_2_. The cells were then maintained in cell culture flasks and passaged every 2–3 days when they reached approximately 80% confluence. The growth medium was aspirated to subculture, the monolayer washed with phosphate‐buffered saline (PBS), and 3 mL of trypsin–EDTA was added. Once detached, the cells were reseeded at a density of 1 × 10^4^ cells/cm^2^ [72].

##### 3.8.2.2 Anti‐Inflammatory Assay

To assess the anti‐inflammatory activity, the secretion of IL‐6 and IL‐8 from HaCaT cells was quantified after treatment with plant extracts and subsequent stimulation with TNF‐α [72]. Cells were seeded in 48‐well plates (40 000 cells/well in 0.4 mL medium) and incubated for 24 h. The following day, supernatants were removed, cells were washed twice with PBS, and new medium containing two concentrations, that is, 100 or 500 µg/mL of the different plant extracts, 10 µM (4.31 µg/mL) budesonide (positive control), or vehicle control (96% ethanol or 100% methanol, each as 0.25% of total volume) was added. TNF‐α (0.4 ng/mL) was included to induce inflammation. After 24 h of incubation, supernatants were collected and frozen at −20°C. Sandwich ELISA (Thermo Fisher Scientific Inc.) was used to quantify IL‐6 and IL‐8 levels, and untreated and unstimulated controls were included. We performed all our experiments in quadruplicate.

##### 3.8.2.3 Cytotoxicity Assay

Cytotoxic effects of the plant extracts were evaluated using the MTT (3‐(4,5‐dimethylthiazol‐2‐yl)‐2,5‐diphenyltetrazolium bromide) assay, which aimed to quantify cellular metabolic activity [72]. After TNF‐α stimulation and treatment with the plant extract, the supernatants were removed, and the cells were washed with PBS. Then, 200 µL of 0.3 mg/mL MTT solution (Thermo Fisher Scientific Inc.) was added to each well, and the plates were incubated at 37°C and 5% CO_2_ for 2 h until purple formazan crystals formed. These were solubilized using a 10% SDS solution, and absorbance was measured at 570 nm using a microplate reader. Budesonide served as an anti‐inflammatory control, while 1% Triton X‐100 was used to induce cell death. All treatments were tested in quadruplicate.

### 3.9 Statistical Analysis

Data were analyzed using one‐way analysis of variance (one‐way ANOVA) followed by Dunnett's post hoc test. Results are presented as mean ± SEM of four replicates. A *p* ≤ 0.05 was considered statistically significant. Graphs were generated using GraphPad Prism Software Version 5 (GraphPad Software Inc.; San Diego, CA, USA).

## 4 Conclusion

This study compares the phytochemical profiles and biological activities of wild and hydroponically grown *C. asiatica*, *J. betonica*, and *C. sumatrensis*, three medicinal plants used for wound healing in Uganda. Hydroponic cultivation achieved higher extraction yields in organic solvents, particularly for *C. asiatica* and *J. betonica*. TLC and HPLC profiles revealed overlapping phytochemical classes, terpenoids, flavonoids, and phenolic compounds in both wild and hydroponic extracts, yet wild samples showed more complex banding patterns and chromatographic peaks. GC–MS volatile profiling confirmed a high degree of overlap in core volatiles across all three species, with *C. asiatica* showing the highest chemical overlap (92.86%), while *C. sumatrensis* and *J. betonica* displayed hydroponic‐exclusive volatiles (14.29% discrepancy), reflecting environment‐specific secondary metabolism. LC–MS/MS analysis further illustrated that metabolites such as kaempferol derivatives, camelliaside A, and katononic acid were conserved in both cultivation systems. Certain compounds, like euscaphic acid and glaucaside C, were unique to wild plants, whereas brahmic acid, asiatic acid and arjunglucoside II appeared exclusively in hydroponic extracts. Alcoholic extracts from both cultivation types showed moderate antibacterial effects against certain bacterial strains, with *C. sumatrensis* demonstrating the strongest activity. Aqueous extracts of wild *C. asiatica* and hydroponic *C. sumatrensis* significantly lowered IL‐6 levels in TNF‐α‐stimulated HaCaT keratinocytes, with no cytotoxicity observed. To the best of our knowledge, no previous study has directly compared the antibacterial or anti‐inflammatory properties of medicinal plants grown hydroponically with those from wild collections. Therefore, our study represents the first effort to make such a comparison. Our results indicate that hydroponic cultivation preserves plant metabolites and may offer a sustainable alternative to wild collection. Although unique compounds can be lost or gained depending on the environment, hydroponics preserves the key phytochemical and bioactivity characteristics necessary for producing standardized phytopharmaceuticals. This approach offers a practical means to secure medicinal plant raw materials while also promoting biodiversity conservation.

## Author Contributions

**Ivan Kahwa**: conceptualization, methodology, validation, formal analysis, investigation, data curation, writing – original draft preparation, writing – review and editing, visualization. **Christina Seel**: validation, formal analysis, investigation, data curation, writing – original draft preparation, visualization. **Hilda Ikiriza**: methodology, formal analysis, investigation. **Maria Kulosa**: methodology, formal analysis, investigation, visualization. **Susan Billig**: methodology, formal analysis. **Claudia Wiesner**: methodology, validation, formal analysis, resources, data curation, writing – review and editing, visualization, supervision. **Anke Weisheit**: resources, supervision, project administration, funding acquisition. Olivia **Harriet Makumbi**: methodology, supervision, project administration, funding acquisition. **André Gerth**: supervision, funding acquisition. **Leonard Kaysser**: leonard, conceptualization, resources, writing – review and editing, visualization, supervision, project administration, funding acquisition. All authors have read and agreed to the published version of the manuscript.

## Funding

The project No. 22.2235.4‐501.00 “Improving the utilization of traditional medicinal plants in Uganda” is part of the German Government and Federal States Programme (German: Bund‐Länder‐Programm, BLP), which is implemented by Deutsche Gesellschaft für lnternationale Zusammenarbeit (GIZ) GmbH on behalf of the Federal Ministry for Economic Cooperation and Development (BMZ). The BMZ finances the project and its procurements. This project is co‐financed by tax funds based on the budget passed by the Parliament of the Free State of Saxony. This research was funded by the European Regional Development Fund (ERDF, Europäischer Fond für Regionale Entwicklung EFRE, “Europe funds Saxony”, grant no. 100195374) and Leipzig University.

## Conflicts of Interest

The authors declare no conflicts of interest.

## Supporting information




**Supporting File 1**: cbdv70863‐sup‐0001‐SuppMat.pdf

## Data Availability

The data that support the findings of this study are available from the corresponding author upon reasonable request.
