# Supplementary material for: Comparative Phytochemical Profiles of Medicinal Plants Used for Wound Treatment: Insights From Wild and Hydroponically Cultivated Species in Lugazi Diocese, Uganda
Source: Chem Biodivers. 2026 Jan 17;23(1):e03018. doi: 10.1002/cbdv.202503018 (PMC12811819; doi:10.1002/cbdv.202503018)
Supplement: Supplementary file 1 — Supporting File 1: cbdv70863‐sup‐0001‐SuppMat.pdf [file CBDV-23-e03018-s001.pdf]

Supporting Information (SI)

Comparative Phytochemical Profiles of Medicinal Plants Used for Wound Treatment: Insights from Wild and Hydroponically Cultivated Species in Lugazi Diocese, Uganda

Ivan Kahwa[1,2,3*], Christina Seel[1], Hilda Ikiriza[3,4], Maria Kulosa[1], Susan Billig[5], Claudia Wiesner[5], Anke Weisheit[3], Olivia Harriet Makumbi[6], André Gerth[7] and Leonard Kaysser[1*]

[1] Institute for Drug Discovery, Department of Pharmaceutical Biology, Faculty of Medicine, Leipzig University, 04317 Leipzig, Germany

[2] Department of Pharmacy, Faculty of Medicine, Mbarara University of Science and Technology, Mbarara P.O. Box 1410, Uganda

[3] Pharm-Biotechnology and Traditional Medicine Centre, Mbarara University of Science and Technology, Mbarara P.O. Box 1410, Uganda

[4] Department of Biology, Faculty of Science, Mbarara University of Science and Technology, Mbarara P.O. Box 1410, Uganda

[5] Institute of Analytical Chemistry, Faculty of Chemistry, Leipzig University, 04103 Leipzig, Germany

[6] Lugazi Rural Finance Development Trust, Namagunga, Lugazi, Uganda

[7] Independent Researcher, Fuerstenweg 8, D-04668 Grimma, Germany

* Correspondence:, ivan.kahwa@medizin.uni-leipzig.de/kahwa@must.ac.ug, leonard.kaysser@uni-leipzig.de

<u>Supplementary file S1</u>

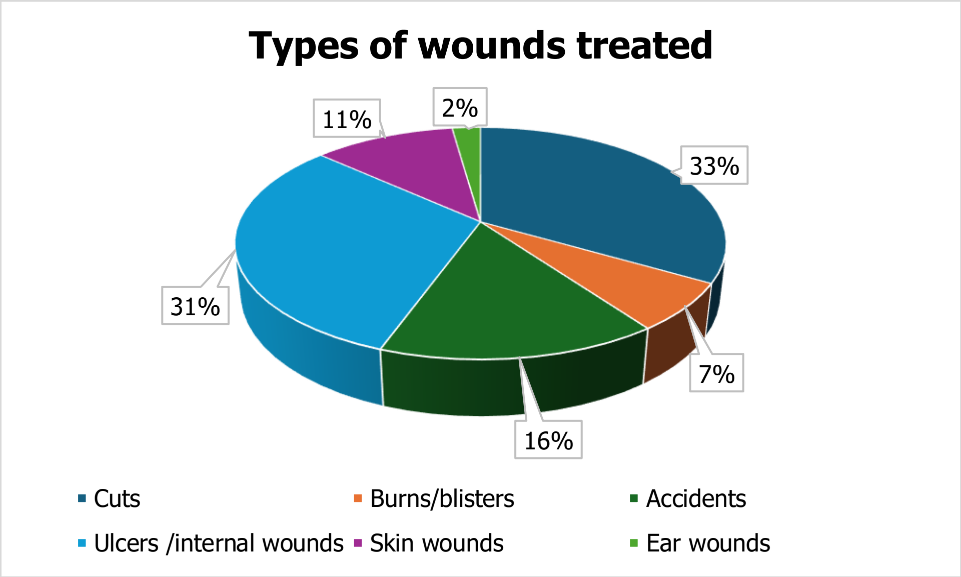

Figure **S1A**Types of wounds treated by the selected medicinal plants in the surveyed villages. Percentages represent the proportion of respondents reporting each wound type (n = 18 participants). Data are descriptive; therefore, no statistical test or P-value applies

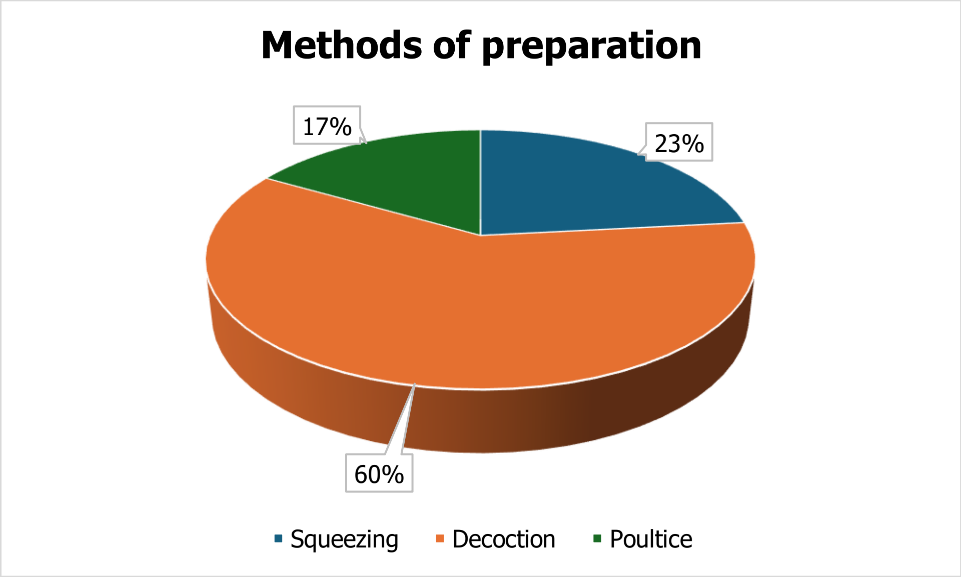

Figure **S1B** Methods of preparation of remedies from the selected medicinal plants. Percentages represent the proportion of respondents reporting each preparation method (n = 18 participants). Data are descriptive; therefore, no statistical test or P-value applies

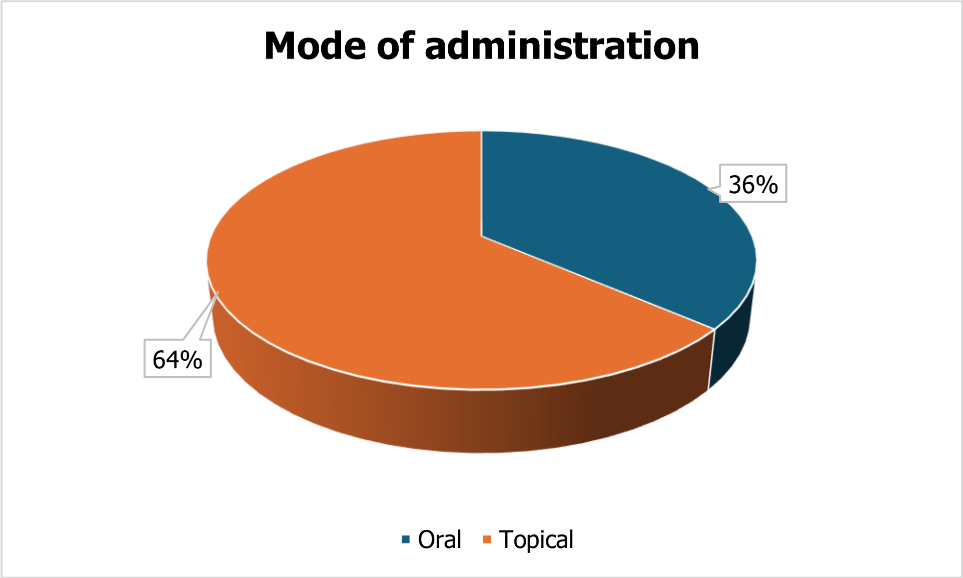

Figure **S1C** Modes of administration of remedies from the selected medicinal plants as reported by respondents. Percentages represent the proportion of each mode (n = 18 participants)

Supplementary file S2

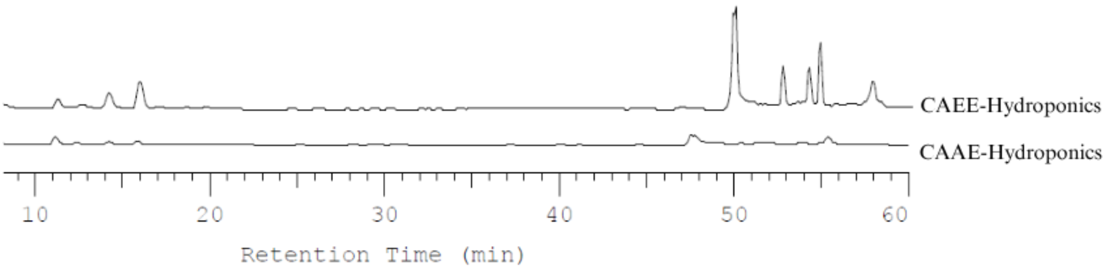

Figure **S2A** HPLC-DAD fingerprints of *Centella asiatica* hydroponic extracts, showing representative chromatographic profiles for CAEE-Hydroponics and CAAE-Hydroponics. Each chromatogram represents a single measurement (n = 1

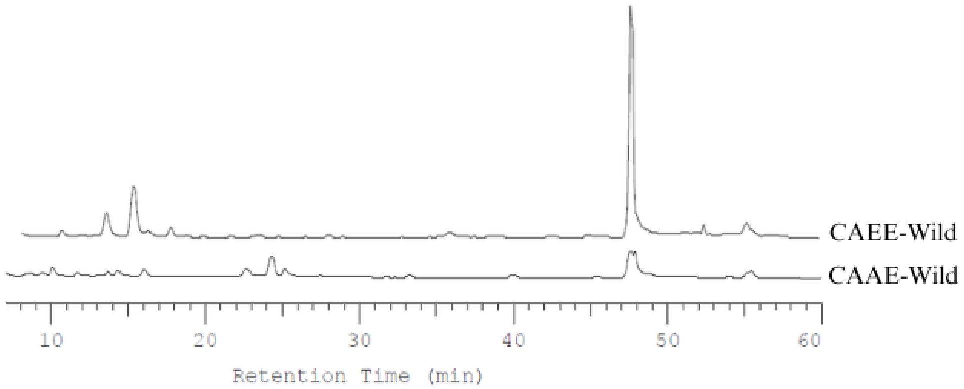

Figure **S2B** HPLC-DAD fingerprints of *Centella asiatica* wild extracts, showing representative chromatographic profiles for CAEE-Wild and CAAE-Wild. Each chromatogram represents a single measurement (n = 1)

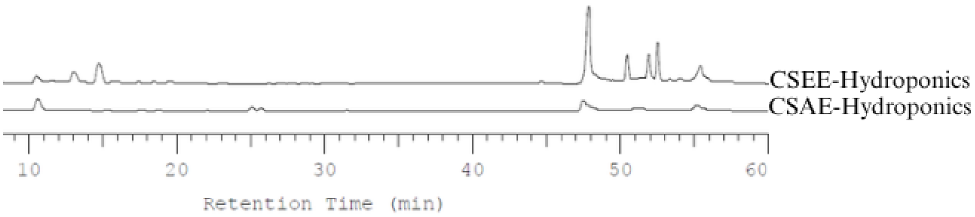

Figure **S2C** HPLC-DAD fingerprints of *Conyza sumatrensis* hydroponic extracts, showing representative chromatographic profiles for CSEE-Hydroponics and CSAE-Hydroponics. Each chromatogram represents a single measurement (n = 1)

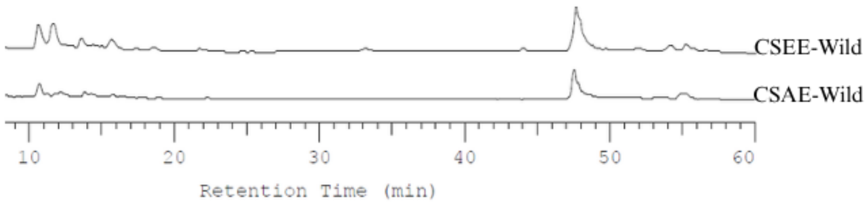

Figure **S2D** HPLC-DAD fingerprints of *Conyza sumatrensis* wild extracts, showing representative chromatographic profiles for CSEE-Wild and CSAE-Wild. Each chromatogram represents a single measurement (n = 1)

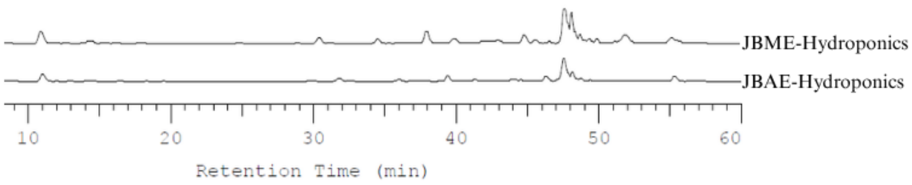

Figure **S2E** HPLC-DAD fingerprints of *Justicia betonica* hydroponic extracts, showing representative chromatographic profiles for JBME-Hydroponics and JBAE-Hydroponics. Each chromatogram represents a single measurement (n = 1)

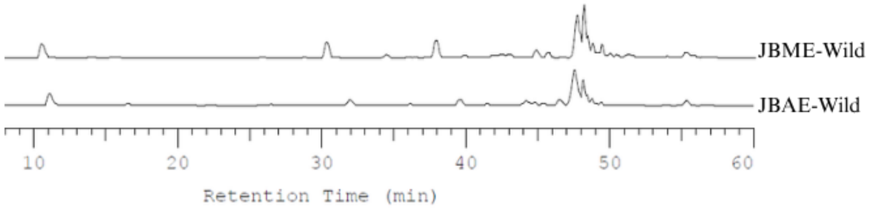

Figure **S2F** HPLC-DAD fingerprints of *Justicia betonica* wild extracts, showing representative chromatographic profiles for JBME-Wild and JBAE-Wild. Each chromatogram represents a single measurement (n = 1)

Supplementary file S3

Table **S3A** GC-MS identified volatile compounds in wild and hydroponically grown *C. asiatica*, *C. sumatrensis*, and *J. betonica* classified by major chemical groups

		Centella asiatica				***Conyza sumatrensis***				***Justicia betonica***			
**Compound name**	**Class**	**Wild**		**Hydroponics**		**Wild**		**Hydroponics**		**Wild**		**Hydroponics**	
		**RT [min]**	**Area**	**RT [min]**	**Area**	**RT [min]**	**Area**	**RT [min]**	**Area**	**RT [min]**	**Area**	**RT [min]**	**Area**
Limonene	Monoterpenes	13.16	2195019	13.16	41168671	13.16	8476331	13.16	754371	13.16	1664347	13.16	811212
α-Pinene		10.09	234257	10.09	362672	10.09	2493603	10.09	438087	-	-	10.081	51397
p-Cymene		13.01	151150	-	-	13.01	1610511	13.01	328068	13.01	70560	13.01	70792
β-Pinene		11.50	1287136	11.50	3308914	-	-	11.50	59911	-	-	-	-
γ-Terpinene		14.09	151498	14.09	72486	14.09	664798	14.10	107016	-	-	-	-
β-*m*yrcene		11.90	142542	11.90	461628	11.91	262714	-	-	11.90	388408	11.91	168108
Sabinene		11.35	62271	11.36	131057	-	-	-	-	-	-	-	-
β-Ocimene		13.72	95001	13.72	287194	-	-	-	-	13.72	206670	13.73	86455
α-Thujol		14.99	114298	14.96	182407	-	-	-	-	14.98	244664	14.98	145522
Linalool		-	-	-	-	-	-	-	-	15.37	2311429	15.38	959991
β-Cyclocitral		18.99	149107	19.00	209693	18.99	165825	-	-	19.00	52468	19.00	53162
Camphene		10.61	37214	10.61	66061	10.61	213035	10.60	73756	-	-	-	-
Cubenene	Sesquiterpenes	24.71	273356	24.71	2090448	-	-	-	-	-	-	-	-
β-Bisabolene		26.45	477767	26.45	2284381	-	-	-	-	-	-	-	-
cis-Calamenene		-	-	-	-	26.70	653231	26.70	279195	-	-	-	-
β-Bourbonene		-	-	-	-	23.62	808608	23.62	163526	-	-	-	-
β-Caryophyllene		-	-	-	-	24.18	112427	24.17	104099	-	-	-	-
α-Selinene		-	-	-	-	26.29	1798674	26.29	582233	-	-	-	-
β-Cubebene		22.62	261170	22.63	3030268	22.62	390514	22.61	80076	-	-	-	-
β-Selinene		26.14	136465	26.14	542061	26.15	2367260	26.15	754200	-	-	-	-
Aristolene		-	-	-	-	24.56	13809011	24.56	2946337	-	-	-	-
α-Ylangene		23.24	67896	23.23	301241	23.23	214234	23.23	94846	-	-	-	-
Daucene		23.47	2057283	23.48	10578040	-	-	-	-	23.48	46118	23.49	21549
α-Copaene		23.40	287495	23.40	1249861	23.40	3695018	23.40	869896	23.40	71021	23.40	100214

β-Copaene		-	-	-	-	25.986	865052	-	-	-	-	-	-
Modhephene		23.58	390767	23.59	4339130	-	-	-	-	-	-	-	-
Selina-5,11-diene		23.91	97852	23.92	537677	-	-	-	-	-	-	-	-
α-Isocomene		23.75	130619	23.75	2184021	23.74	1887809	23.74	621354	-	-	23.75	62613
Caryophyllene		24.56	1317541	24.57	16573215	-	-	-	-	24.56	175156	24.56	232737
δ-cadinene		26.64	210948	26.65	2124855	26.65	818943	26.65	490140	26.65	48041	26.65	29360
γ-Cadinene		26.57	182021	26.57	1139964	26.57	538824	26.57	186209	-	-	-	-
trans-Cadina-1,4-diene		-	-	-	-	26.90	492350	26.90	353158	-	-	-	-
γ-Muurolene		25.54	2339511	-	-	25.85	1878672	25.85	506212	-	-	-	-
α-Muurolene		26.30	195474	26.30	1873526	26.96	317274	26.96	121987	-	-	-	-
α-Calacorene		-	-	-	-	27.03	877023	27.02	220921	-	-	-	-
1(10),11-Eremophiladien-9-ol		-	-	-	-	27.14	145732	27.13	39733	-	-	-	-
α-humulene		25.45	502933	25.45	1830127	25.45	5905200	25.45	1485164	25.45	96755	25.45	149008
α-Bergamotene		24.86	2219470	24.87	14081566	-	-	-	-	24.88	71804	24.86	54246
γ-Amorphene		26.20	106703	26.20	1513105	-	-	-	-	-	-	26.21	42307
Spathulenol		27.57	1287652	27.57	9349835	-	-	-	-	-	-	27.58	22831
α-Bulnesene		26.37	181240	26.37	1221382	23.49	112849	23.47	45554				
δ-Elemene		-	-	-	-	22.293	39233	22.293	48158	-	-	-	-
α-Curcumene		25.94	616089	25.94	2817865	-	-	-	-	-	-	-	-
β-Patchoulane		27.99	70518	27.98	729180	24.86	480311	-	-	-	-	-	-
β-trans-Bergamotene		-	-	-	-	25.31	2536732	25.30	403903	-	-	-	-
Acora-2,4(15)-diene		25.17	374882	25.18	2030785	-	-	-	-	-	-	-	-
β-Farnesene		25.30	7795525	25.31	56171084	-	-	-	-	-	-	-	-
Guaia-6,9-diene		24.96	694567	24.96	3125650	-	-	-	-	-	-	-	-
6,10-Epoxy-7(14)-isodaucene		-	-	-	-	27.46	126584	27.46	66863	-	-	-	-
Caryophyllene oxide		27.64	541304	27.64	4140332	27.64	901032	27.64	687637	-	-	-	-
Salvial-4(14)-en-1-one		27.81	223505	27.80	1606507	27.80	938412	27.80	375097	-	-	-	-
Guaia-3,10(14)-diene<9,11-epoxy->		-	-	-	-	28.08	627232	28.08	148320	-	-	-	-
Aristola-1(10),8-diene		-	-	-	-	28.23	96517	28.24	56202	-	-	-	-
α-Costol		-	-	-	-	28.57	688926	28.57	380726	-	-	-	-
Cadalene		-	-	-	-	28.75	564648	28.76	132970	-	-	-	-
Sesquisabinene		26.72	262338	26.72	2264092	-	-	-	-	-	-	-	-
β-elemene		31.32	67888	-	-	-	-	-	-	-	-	-	-
Neophytadiene	Diterpene	30.23	2450627	30.23	25385884	30.23	14226359	30.23	391996	30.23	102238	-	-
Butanal	Aldehydes	2.61	1079305	2.62	1285461	-	-	-	-	-	-	-	-
Isopentanal		3.27	1297955	3.28	493604	-	-	-	-	-	-	-	-
α-Methylbutanal		3.39	1190613	3.39	1487262	3.40	368681	3.38	220234	3.37	377207	3.38	120387
Pentanal		3.87	646941	3.89	692826	3.87	1599744	3.87	391015	3.87	287002	3.86	453597
Hexanal		6.11	1071544	6.13	587860	6.11	7569384	6.11	3313154	6.13	190255	6.11	409564
Benzaldehyde		11.01	331911	11.01	208665	11.01	208367	11.02	109161	-	-	11.01	144341
3-Heptanone	Ketones	8.62	138375	-	-	-	-	8.62	54165	8.60	237684	8.60	55137
3-Octanone		12.75	153948	12.76	95838	12.77	87915	-	-	12.79	81385	12.74	58633

Methylbutenol	Alcohols	2.79	789011	2.80	777698	-	-	2.77	304785	2.78	414714	2.77	75848
2-Methyl-1-propanol		2.94	348224	2.95	535356	-	-	-	-	2.94	382312	2.94	100968
1-Penten-3-ol		3.65	548690	3.67	408496	3.66	906525	3.66	262803	3.67	139726	3.66	73841
trans-3-Hexen-1-ol		7.71	910457	7.70	298963	7.66	663108	7.70	110814	7.70	1055947	7.70	3413307
Hexanol <n->		8.18	66045	8.14	129454	-	-	-	-	8.12	915598	8.11	844868
3,7,11-Trimethyl-1-dodecanol		14.65	506797	14.66	771510	-	-	-	-	14.66	538613	14.66	490843
3,4-Dimethylcyclohexanol		15.67	305017	15.68	130298	15.68	211955	15.58	165321	15.68	71946	15.68	173549
p-Menthane-1,3-diol		16.63	223682	16.64	167725	16.64	158426	-	-	16.64	90578	16.63	148857
2,4-Dimethylheptane	Hydrocarbons	6.67	47406	6.69	107309	-	-	-	-	-	-	-	-
Octane <n->		7.93	283451	7.93	44872	7.931	291939	-	-	7.93	130961	7.89	187013
Undecane <n->		11.14	91602	11.13	62879	11.13	95634	11.14	28160	-	-	11.13	25686
Decane <n->		12.256	51684	12.26	126233	12.25	155643	-	-	12.24	149454	12.26	52909
3,3-Dimethyloctane		12.47	458049	12.47	615771	12.48	788693	12.47	263252	12.47	537155	12.47	338141
4-Methyldecane		12.61	412176	12.61	587646			12.61	348832	12.61	516057	12.61	416957
2,5-Dimethylnonane		12.83	192601	12.84	206292	12.84	306013	12.84	101290	12.84	146134	12.84	172053
Dodecane <n->		12.95	139450	13.00	629258	-	-	-	-	12.95	120343	12.95	108120
3,7-Dimethyldecane		14.17	226574	14.16	223893	-	-	14.16	181669	14.17	190721	14.16	172119
2,4-Dimethyl 1-decene		14.79	508404	14.79	765128	14.79	987096	14.79	415938	14.79	533992	14.79	526841
5-(1-Methylpropyl)nonane		15.17	145391	15.17	185456	15.18	120272	15.18	59680	15.17	150455	15.17	91644
2,6,6-Trimethyldecane		15.88	78623	-	-	15.88	127739	15.87	109541	15.89	132051	15.89	141448
Tridecane <n->		16.22	24634	16.23	60532	16.23	66819	16.23	30042	-	-	16.22	210832
3-Methylcyclopentyl acetate	Esters	9.04	229830	9.04	149910	-	-	-	-	-	-	-	-

Table **S3B** LC-MS/MS-based annotation of secondary metabolites in wild and hydroponically grown *Centella asiatica*

**Compound**	**Molecular formula**	**Class**	**Fragmentation ions** ***m/z***	***Centella asiatica***			
				**Wild**		**Hydroponics**	
				**RT [min], ion mode**		**RT [min], ion mode**	
				Aq	EtOH	Aq	EtOH
Quercetin-3-O-glucuronoside	$C_{21}H_{18}O_{13}$	Flavonoids	253,301,325	4.95, [M - H]-,[M + H]+	4.96,[M + H]-,	4.94, [M - H]-	
Quercetin	$C_{15}H_{10}O_7$	Flavonoids	151		5.16, [M-H]-		
Kaempferol	$C_{15}H_{10}O_6$	Flavonoids	153, 285,287	9.73, [M- H]-	5.70, [M + H]+<br>9.77, [M - H]-		9.70, [M + H]+<br>9.77, [M - H]-
Euscaphic Acid	$C_{30}H_{48}O_5$	Triterpenoids	187, 189, 201,205	7.99, [M + H]+			
Medicagenic acid	$C_{30}H_{46}O_6$	Triterpenoids	503	10.27,[M + H]+			
3-hydroxytetracosanoic acid	$C_{24}H_{48}O_3$	Diterpenoids	337,383			26.86, [M - H]-	
Asiatic Acid	$C_{30}H_{48}O_5$	Triterpenoids	511				12.77,[M + H]+<br>12.86,[M - H]-
Brahmic Acid	$C_{30}H_{48}O_6$	Triterpenoids	133 159, 187,189,199,201,215, 217,501				11.05,[M + H]+<br>11.07,[M - H]-
Kaempferol 7-O-glucoside	$C_{21}H_{20}O_{11}$	Flavonoids	284,285				5.64, [M - H]-

Table **S3C** LC-MS/MS-based annotation of secondary metabolites in wild and hydroponically grown *Justicia betonica*

**Compound**	**Molecula r formula**	**Class**	**Fragmentation ions**	***Justicia betonica***	
				**Wild**	**Hydroponics**
				**RT [min], ion mode**	**RT [min], ion mode**

				Aq	MetOH	Aq	MetOH
Camelliaside A	$C_{33}H_{40}O_{20}$	Flavonoids	284, 285, 287	3.88,[M + H]+ 3.84, [M-H]-	3.87,[M + H]+ , [M - H]-	3.82,[M-H]- 3.86,[M + H]+	
Glaucaside C	$C_{42}H_{68}O_{14}$	Triterpene saponins	437,455	8.75,[M + H]+			
Katononic Acid	$C_{30}H_{46}O_{3}$	Triterpenoids	187, 197, 205,217, 235, 285, 437,455,	7.79,[M + H]+	7.11,[M + H]+	7.78,[M + H]+	7.11,[M + H]+
Pentacosanedioic acid	$C_{25}H_{48}O_{4}$				21.99, [M - H]-		
Quercetin 3-(3R-Glucosylrutinoside)	$C_{33}H_{40}O_{21}$	Flavonoids	300, 303		3.32,[M + H]+, [M - H]-		
Kaempferol-3-O-rutinoside	$C_{27}H_{30}O_{15}$	Flavonoids	284,285,287,331		5.04,[M + H]+,[M - H]-	4.99,[M - H]- 5.03,([M + H]+)	5.01,[M - H]-
Arjunglucoside II	$C_{36}H_{58}O_{10}$	Triterpenoids	603,647			10.92,[M - H]-	
Queretaroic Acid	$C_{30}H_{48}O_{4}$	Triterpenoids	145, 159,187,201,217,231,297		9.31,[M + H]+		
Oleanonic Acid	$C_{30}H_{46}O_{3}$	Triterpenoids	145,,147,149,159, 175,187,189,201,205,235,285,437		11.99,[M + H]+	7.09, [M + H]+	

Table **S3C** LC-MS/MS-based annotation of secondary metabolites in wild and hydroponically grown *Conyza sumatrensis*

**Compound**	**Molecular formula**	**Class**	**Fragmentation ions**	***Conyza sumatrensis***			
				**Wild**		**Hydroponics**	
				**RT [min], ion mode**		**RT [min], ion mode**	
				Aq	EtOH	Aq	EtOH
Luteolin 7-O-diglucuronide	$C_{27}H_{26}O_{18}$	Flavonoid	285,351	3.66,[M - H]-			
Chrysoeriol 7-O-diglucuronide	$C_{28}H_{28}O_{18}$	Flavonoid	351	4.54, [M - H]-			
Flavonoid O-glucuronide	$C_{23}H_{16}O_{12}$	Flavonoid	295	4.95, [M-H]-			
Acetylated luteolin 7-O-glucuronide	$C_{23}H_{20}O_{13}$	Flavonoid	285	5.52, [M-H]-			

4″-O-acetylglucuronide of kaempferol	$C_{23}H_{20}O_{13}$	Flavonoid	285	5.93,[M - H]-			
Flavonoid-7-O-glucuronide	$C_{23}H_{20}O_{12}$	Flavonoid	269		7.29,[M-H]-		
Triterpene saponin	$C_{37}H_{60}O_{11}$	Prenol lipids	633		9.74,[M-H]-		
Diterpene glycoside	$C_{46}H_{76}O_{13}$	Prenol lipids	277,293		22.56,[M-H]-		
Diterpenoid	$C_{28}H_{52}O_{7}$	Prenol lipids	187, 255		22.87,[M-H]-		
Diterpenoid	$C_{30}H_{56}O_{7}$	Prenol lipids	461		23.81,[M-H]-		
Diterpenoid	$C_{23}H_{46}O_{3}$	Prenol lipids	323		25.84,[M-H]-		
11,13-dihydroxytetracos-9-enoic acid	$C_{24}H_{46}O_{4}$	Prenol lipids	339			19.93,[M-H]-	
Octacosanedioic acid	$C_{28}H_{54}O_{4}$	Prenol lipids	407, 451			22.81,[M-H]-	
Diterpenoid	$C_{30}H_{56}O_{7}$	Prenol lipids	461				23.85,[M-H]-

Supplementary file S4

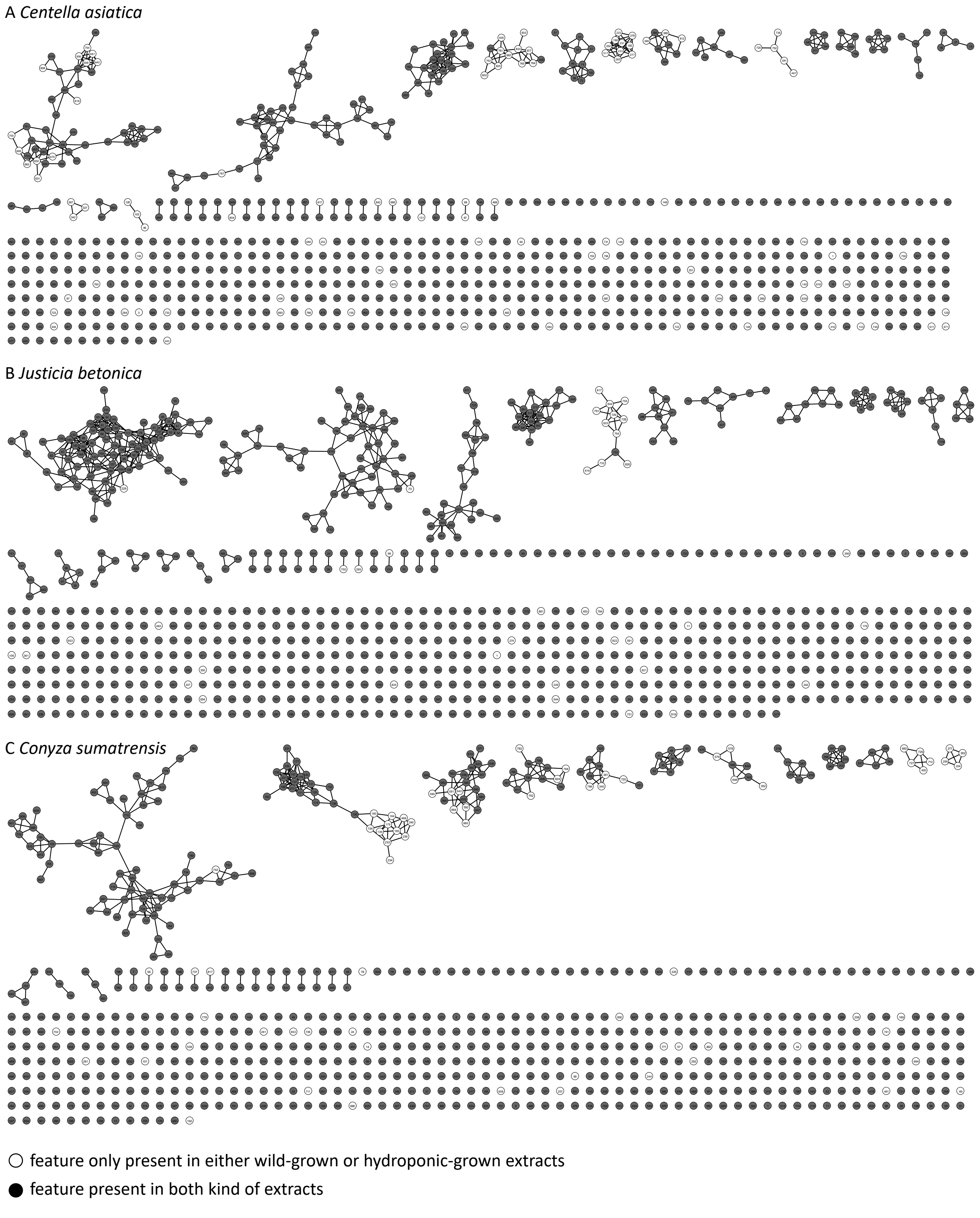

Figure **S4**: Feature-based molecular networks of (A) *Centella asiatica*, (B) *Justicia betonica*, and (C) *Conyza sumatrensis*, illustrating the distribution of detected features in wild-grown versus hydroponic-grown extracts. Each node represents an MS/MS feature, with filled circles indicating features present in both growth conditions and open circles indicating features unique to either wild-grown or hydroponic-grown samples. All singleton nodes are annotated with their corresponding feature ID. Data represent a single analysis per extract (n = 1); therefore, no statistical test or P-value applies

Supplementary file S5

Anti-inflammatory assays

a) Interleukin 6

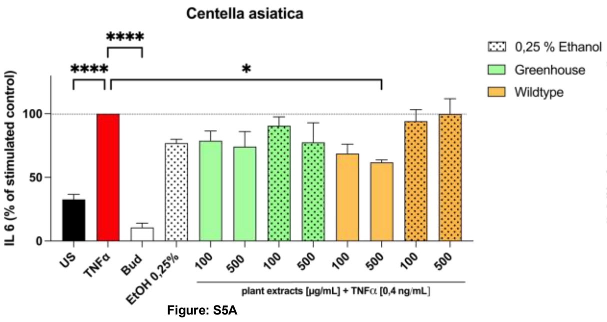

Figure: S5A

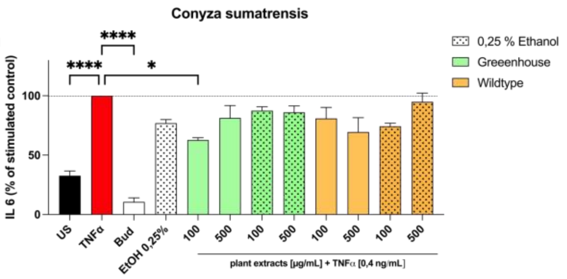

Figure: S5B

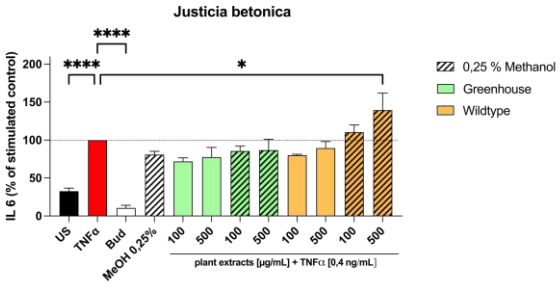

Figure: S5C

Figure S5A-S5C (IL-6 release): Effects of wild-type and hydroponic plant extracts on TNF-α–induced IL-6 release in HaCaT keratinocytes. (S4A) *Centella asiatica*: The aqueous wild extract (500 µg/mL) significantly reduced IL-6 secretion by approximately 45% ($p < 0.01$) compared to the TNF-α control, while the hydroponic aqueous extract (100 µg/mL) reduced IL-6 by about 30% ($p < 0.05$); ethanolic extracts showed no significant effect. (S4B) *Conyza sumatrensis*: The aqueous hydroponic extract (100 µg/mL) decreased IL-6 release by approximately 40% ($p < 0.05$), whereas the wild extract (500 µg/mL) showed no significant inhibition. (S4C) *Justicia betonica*: The methanolic wild extract (500 µg/mL) increased IL-6 secretion by about 35% ($p < 0.05$) compared to TNF-α control, while hydroponic extracts exhibited no significant effect. Budesonide (10 µM) served as the positive control, reducing IL-6 levels by approximately 70% in all assays. Data represent mean ± SEM, n = 4.

b) Interleukin 8

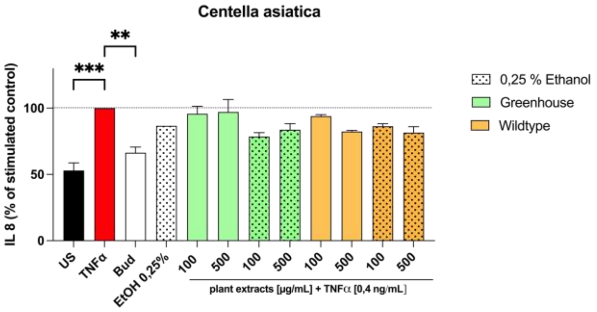

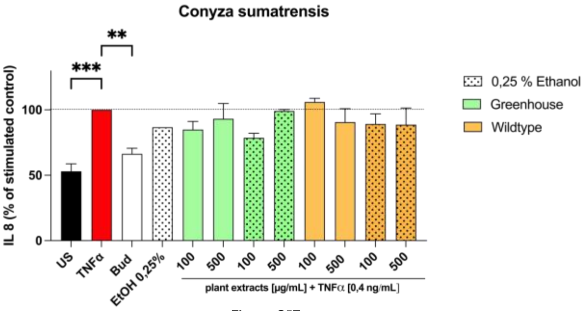

Figure: S5D

Figure: S5E

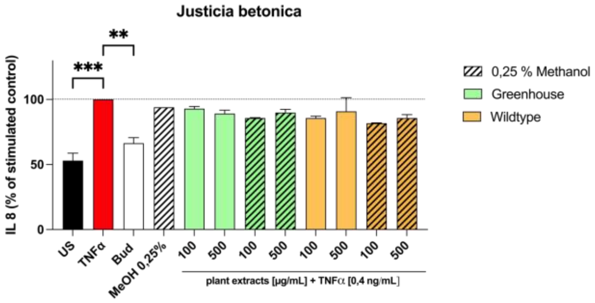

Figure: S5F

Figures S5D–S5F (IL-8 release): None of the plant extracts significantly altered IL-8 secretion at 100–500 µg/mL. Budesonide reduced IL-8 by ~65 %. n = 2 per condition.

Cytotoxicity assay

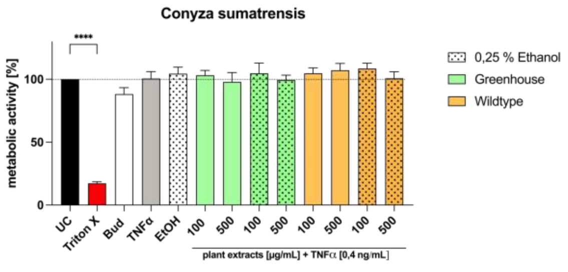

Figure: S5G

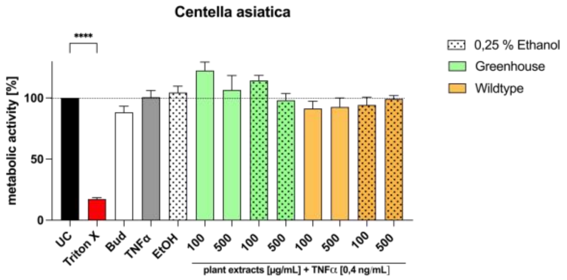

Figure: S5H

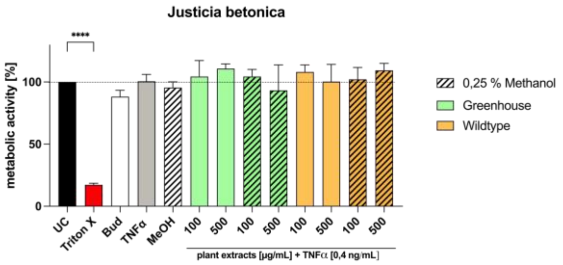

Figure: S5I

Figures S5G–S4I (Cytotoxicity): All plant extracts maintained at least 90% cell viability up to 500 µg/mL compared to the untreated control (100% viability). There was no significant difference between wild and hydroponic sources. Budesonide and Triton X served as positive and cytotoxicity controls, respectively. n = 4, mean ± SEM.
